# On viscoelastic drop impact onto thin films: axisymmetric simulations and experimental analysis

**DOI:** 10.1038/s41598-023-38235-1

**Published:** 2023-07-07

**Authors:** M. R. Rezaie, M. Norouzi, M. H. Kayhani, S. M. Taghavi, Mirae Kim, Kyung Chun Kim

**Affiliations:** 1 Faculty of Mechanical Engineering, Shahrood University of Technology, Shahrood, Iran; 2 Chemical Engineering Department, Laval University, Quebec, G1V 0A6 Canada; 3 School of Mechanical Engineering, Eco-friendly Smart Ship Parts Technology Innovation Center, Pusan National University, Busan, 46241 Republic of Korea; 4 Rolls-Royce and Pusan National University Technology Center, Pusan National University, Busan, Republic of Korea

**Keywords:** Engineering, Physics

## Abstract

This study investigates the effect of fluid elasticity on axisymmetric droplets colliding with pre-existing liquid films, using both numerical and experimental approaches. The numerical simulations involve solving the incompressible flow momentum equations with viscoelastic constitutive laws using the finite volume method and the volume of fluid (VOF) technique to track the liquid’s free surface. Here, the Oldroyd-B model is used as the constitutive equation for the viscoelastic phase. Experiments are also performed for dilute viscoelastic solutions with 0.005% and 0.01% (w/w) polyacrylamide in 80:20 glycerin/water solutions, in order to ensure the validity of the numerical solution and to investigate the elasticity effect. The formation and temporal evolution of the crown parameters are quantified by considering the flow parameters, including the fluid’s elasticity. The results indicate that the axisymmetric numerical solutions reasonably agree with the experimental observations. Generally, the fluid’s elasticity can enlarge the crown dimension at different thicknesses of the fluid film. Moreover, at intermediate values of the Weissenberg number, the extensional force in the crown wall can control the crown propagation. Furthermore, the results reveal that the effects of the Weber number and the viscosity ratio on this problem are more significant at higher values of the Weissenberg number.

## Introduction

The dynamics of the crown formation due to the impact of a drop onto a thin liquid film is of interest in fluid mechanics and engineering applications. The drop impact on liquid film has several significant applications, particularly in inkjet printing, coating, spraying, painting, and cooling processes. The complexity of the drop impact dynamic makes this phenomenon important. Many studies^1–6^ have investigated the drop impact onto the liquid film problem, from different perspectives, mainly focusing on Newtonian fluids. In Newtonian fluids, the regimes of the drop impact on liquid film have been classified by Manzello and Yang^7^ and Rein^8,9^. The studies on the drop impact on a liquid film by Rein^8^, Cossali et al.^10^, Wang and Chen^11^, and Rioboo et al.^12^ have revealed that the crown in Newtonian fluids is formed at large impact Weber numbers and large drop velocities.

Over the last few decades, various theoretical, numerical, and experimental works have analyzed the impact phenomenon in Newtonian fluids. Numerical simulation is an alternative method to model complex two-phase flows. In a numerical simulation, the tracking of the interface deformation between the phases is critical and can be treated by different numerical techniques, for example, the Marker and Cell (MAC) method, the volume of fluid (VOF) technique, the lattice Boltzmann method (LBM), the level-set method (LSM), etc. For instance, Yarin and Weiss^13^ have theoretically introduced the concept of a kinematic discontinuity. In this study, the results show that the crown radius varies exponentially with time. Roisman and Tropeal^14^ have theoretically developed a model for the general case of the drop impact on a wetted surface while ignoring surface tension and viscous forces. They have shown their model to be suitable for high-impact velocities, low viscosities, and thin liquid films, and their results agree with previous experimental studies. Also, they have considered the shape of the crown geometry in the case of an oblique impact and the interaction of two drops’ impact onto a liquid film. Trujillo and Lee^15^ have analytically and numerically modeled the crown formation in a droplet splashing problem. They have introduced a model for propagating the crown formed during the droplet impact. They have presented their results in both viscid and inviscid situations, while their model is also suitable for considering a drop's splashing on a thin fluid film. Cossali et al.^16^ have experimentally represented the time evolution of the crown geometrical parameters and the secondary droplet size. Their results have indicated that the crown shape strongly depends on the surface tension, but the film thickness does not significantly affect the crown characteristics. The experimental investigation of the drop impact on a liquid film by Levin and Hobbs^17^ has revealed that the crown radius has a square root dependence on time. This relation has also been reported by Cossali et al.^16^. Okawa et al.^18^ have experimentally studied the splashing thresholds during the drop impact onto a fluid film. They have used different fluids to investigate the effects of the critical Weber number and the fluid film thickness on the splashing thresholds.

Josserand and Zaleski^19^ have numerically investigated the transition between the splashing and the deposition for the droplet impact on a thin liquid film. Their results have shown that, at early instances of the drop impact, a square root relation is observed between the crown radius and time, while the viscosity shows no meaningful effect on the crown evolution. Mukherjee and Abraham^20^ have numerically investigated the crown shape in the drop impact on a liquid film using a high-density ratio lattice Boltzmann model. They have found that, on thin films, the crown radius and height growth rates have the same trend with increasing the film thickness; however, they have reported an opposite trend for thicker films. Furthermore, an increase in the surrounding gas density results in a decrease in the growth rate of the crown dimension, while the gas viscosity has a minor effect on the crown height and radius. Coppola et al.^2^ have performed numerical simulations on a two-dimensional drop impact on a thin film. They have simultaneously employed the Navier–Stokes equations and the VOF method to solve the problem while considering viscous, inertia, and surface tension forces in the governing equations. They have presented the results of their simulations for early and intermediate instants of the impact. Their simulations quantifies the velocity, pressure fields, and interface shapes for different parameters. They have emphasized that the impact problem is essentially three-dimensional, and the axisymmetric assumption is unacceptable when the instability occurs in the circular lamellae. Both viscous and inviscid assumptions have been considered in analytical and numerical solutions in previous works, taking into account the effects of the film thickness, the droplet velocity, and the wall friction on the crown dynamics in Newtonian fluids.

However, the effects of the fluid’s rheological properties, especially the elasticity and the nonlinear viscosity, have so far received less attention in the literature^21,22^. Lampe et al.^23^ have experimentally investigated the water drop impact on viscoelastic wormlike micelle polymer solutions. They have studied the effects of the viscoelastic fluid properties and its compositions as well as the fluid film thickness, on the progress of the crown shape, the formation of satellite droplets, and the evolution of the Worthington jet. They have introduced a correlation for the onset of splashing of secondary droplets for a thick film of the viscoelastic fluid. Tome et al.^24,25^ have numerically investigated the viscoelastic drop impact on the same liquid pool. They have employed the MAC technique to simulate three-dimensional free surface flows, presenting results for different Reynolds and Weissenberg numbers while neglecting the effect of the surface tension force. Their simulations have emphasized the significant effect of viscoelasticity on the two-phase flow. Izbassarov et al.^26^ have developed a numerical solver for suspending rigid and deformable particles in viscoelastic fluids. They have employed different methods to specify the interface between the phases involved while validating their solver for different cases of non-Newtonian fluids’ single- and two-phase flows. Recently, Rezaie et al.^27,28^ have numerically studied the impact of a two-dimensional (plane) drop onto a pre-existing liquid film in a viscoelastic fluid. In their work, they have applied the finite volume method (FVM) and the VOF technique to solve the governing equations, which included a non-linear constitutive equation. In their analysis, they have considered the elastic and surface tension forces, finding that the viscoelasticity has a major influence on the formation and temporal evolution of the crown shape.

The present work investigates, numerically and experimentally, the effect of fluid’s elasticity, and extensional viscosity on axisymmetric drop impacts onto pre-existing liquid films, with the following novelties. Firstly, compared to the two-dimensional plane analysis^27^, our axisymmetric simulations provide more realistic and precise results. Secondly, by conducting accompanying experiments, the validity of the present numerical simulations is examined, while a deeper insight is gained into the effects of the flow parameters. In the experimental part, two Boger fluids are used as the viscoelastic phase to investigate the effects of rheological properties on the drop impact onto fluid films. In the numerical part, the Oldroyd-B model is used as the constitutive equation for the viscoelastic phase, and the surrounding air is considered a Newtonian fluid. The FVM and VOF techniques are used to solve the governing equations. Finally, the effects of the main flow parameters, including the Weissenberg number, the viscosity ratio, the film thickness, and the Weber number, on the evolution of the crown are investigated in detail.

## Experiment observation

**Figure 1:**
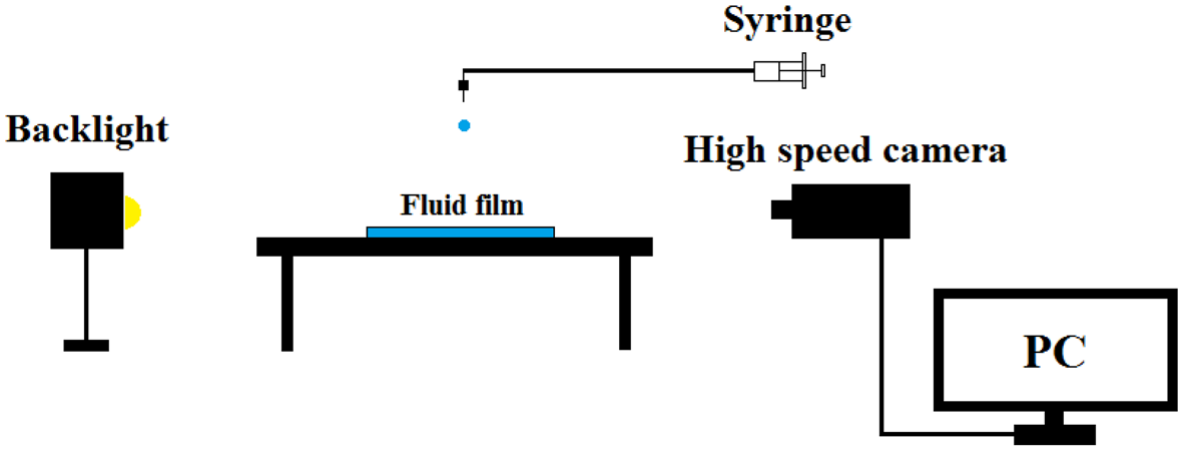
Schematic illustration of the experimental setup.

The schematic illustration of the experimental setup is presented in Fig. 1. The visualization tests were performed at the air-conditioned room temperature (25 °C). The drop impact on fluid films is recorded using a high-speed camera (PCO-dimax HS-1) at 8000 frames per second. The Boger fluid is added to 50 × 50 × 1 (mm^3^) and 50 × 50 × 2 (mm^3^) Plexiglas cells for different thicknesses of the fluid film. A syringe is used to release the fluid from an appropriate needle, producing a drop of different sizes. Also, backlight projectors are used to capture high-quality pictures with a distinguishable boundary.

In this study, viscoelastic Boger solutions are made by dissolving 50 ppm (0.005 wt%) and 100 ppm (0.01 wt%) of polyacrylamide (*M*_*w*_ = 5 × 10^6^ g/mol) in 20/80 ($\upsilon /\upsilon$) ratio of de-ionized water/glycerin, denoted as B1 and B2, respectively. The Newtonian fluids based on water/glycerin solutions are denoted as WG1, WG2, and WG3. The viscoelastic solutions are prepared by mixing the mentioned amount of polymer powders into de-ionized water, and stirred for about 8 h with a magnetic stirrer at the temperature of 25 °C. Afterward, glycerin is added to the solution gradually. At last, the resulting solution is mixed for 16 h. It is worth noting that the surface tension coefficient and the viscosity of fluids are measured using a pendant drop IFT tensiometer and a Brookfield viscometer, respectively. Table 1 presents the physical properties of our Newtonian and Boger fluids. The viscosities were measured using an Anton-Paar MCR 302 rheometer. The measuring system used is a cone and plate spindle with a diameter of 50 mm. The viscosity of polymeric fluids (i.e., B1 and B2 samples) is constant, denoting the Boger liquids’ response in the steady shear test.

**Table 1:** Physical properties of fluids at 25 °C.

	$\rho$ [kg/m^3^]	$\sigma \times$ 10^–3^ [N/m]	$\overline{\eta }$ [mPa s]
WG1	1196	65	78.1
WG2	1210	64.5	98.8
WG3	1199	64.5	64.4
B1	1199	64	78
B2	1199	64	98.9

**Figure 2:**
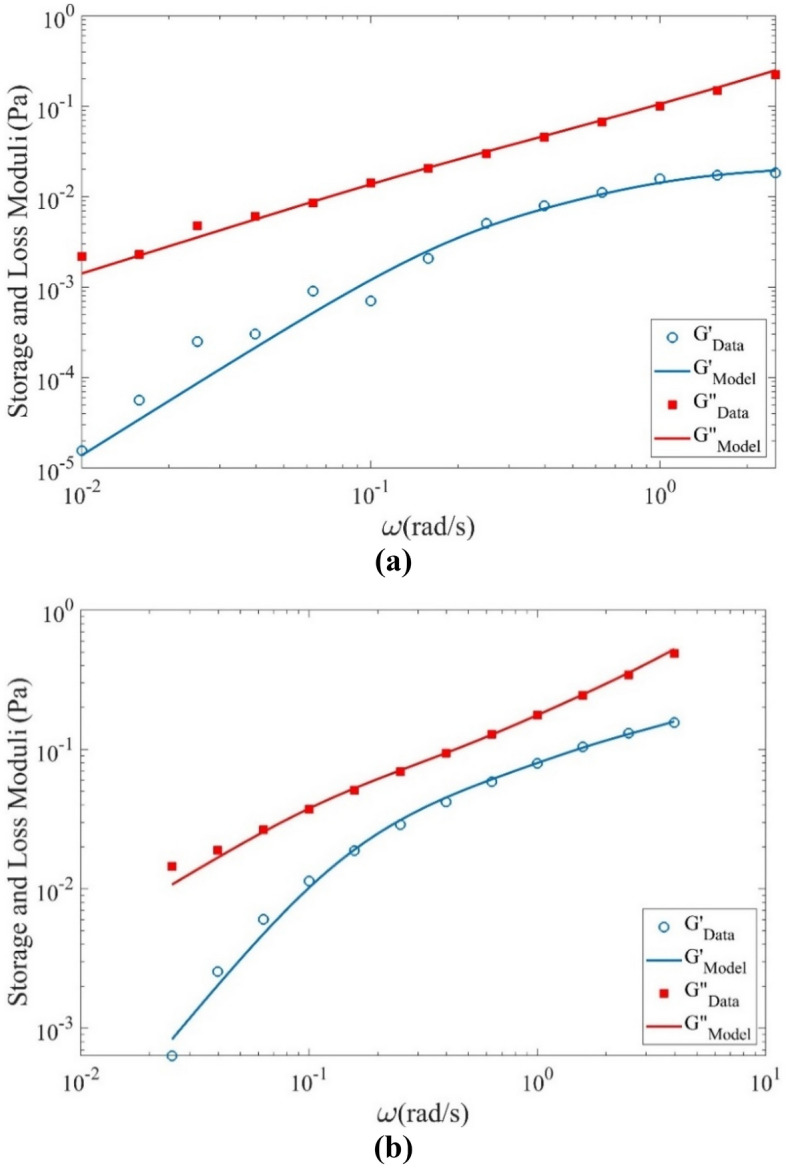
Variation of storage and loss moduli ($G^{\prime}$ and $G^{\prime\prime}$) with angular frequency for (**a**) B1 and (**b**) B2 at 25 °C.

Relaxation time is one of the most important parameters in viscoelastic fluid flows. To estimate the relaxation time, the oscillatory shear tests are employed to obtain $G^{\prime}$(storage) and $G^{\prime\prime}$(loss) moduli. Similar to the viscosity measurement, these data are also obtained using an Anton-Paar MCR302 rheometer. The amplitude sweep tests are performed at a small frequency (1 Hz) to obtain the linear response range. The results indicate that $G^{\prime}$ and $G^{\prime\prime}$ are constant up to 40% strain. In the frequency sweep test, a fixed 10% strain is considered to have a linear set of data, and the frequency range is 0.05–10 Hz to avoid the inertia effect of the measuring system. The results of this test are shown in Fig. 2. The multi-mode Maxwell constitutive equation is used to interpret the data of the frequency sweep test. The storage and loss modulus of this model in the oscillatory shear test are as follows^29^:

1 $$G^{\prime} = \sum\limits_{i = 1}^{n} {\frac{{\eta_{i} \lambda_{i} \omega^{2} }}{{1 + \lambda_{i}^{2} \omega^{2} }}}^{{}} ,$$

2 $$G^{\prime\prime} = \eta_{s} \omega + \sum\limits_{i = 1}^{n} {\frac{{\eta_{i} \omega }}{{1 + \lambda_{i}^{2} \omega^{2} }}}^{{}} ,$$

where $\eta_{s}$ is the viscosity of the Newtonian solvent, $\eta_{i}$ and $\lambda_{i}$ are the viscosity and the relaxation time of each mode, $\omega$ is the frequency and $n$ is the number of modes.

The $G^{\prime}$ and $G^{\prime\prime}$ moduli (Eqs. (1) and (2)) is fitted on the data of the oscillatory test. A MATLAB-based genetic algorithm code is applied to minimize the following error function:

3 $$f = \frac{1}{m}\sum\limits_{i = 1}^{m} {\left[ {\left( {1 - \frac{{G^{\prime}_{Model} (\omega )}}{{G^{\prime}_{Data} (\omega )}}} \right)^{2} + \left( {1 - \frac{{G^{\prime\prime}_{Model} (\omega )}}{{G^{\prime\prime}_{Data} (\omega )}}} \right)^{2} } \right]} ,$$

Table 2 represents the spectrum of the material moduli of the 4-mode Maxwell model. Here, the viscosity of the Newtonian solvent, $\eta_{s}$, is 67 mPa s. Remember that the solvent is a mixture of 80:20 Glycerol and water; this value is its viscosity at 25 °C. The average value of the relaxation time can be obtained as follows:

4 $$\overline{\lambda } = \frac{{\sum {\eta_{i} \lambda_{i} } }}{{\sum {\eta_{i} } }}.$$

**Table 2:** Spectrum of the material moduli for our Boger fluids at 25 °C.

*i*	B1		B2	
	$\lambda_{i}$ [s]	$\eta_{i}$ [Pa s]	$\lambda_{i}$ [s]	$\eta_{i}$ [Pa s]
1	0.0018	0.0145	0.0329	0.0573
2	0.0047	0.0161	0.6303	0.0509
3	1.1539	0.0158	2.4850	0.0807
4	4.2328	0.0284	6.3360	0.1762

According to Table 2 and Eq. (4), the average value of the relaxation time for B1 and B2 are 1.82 s and 3.70 s, respectively. Here, the optimal values of *f* for B1 and B2 are 0.128 and 0.024, respectively. Therefore, the frequency sweep test rheological data modeling via the multi-mode Maxwell model is accurate.

**Figure 3:**
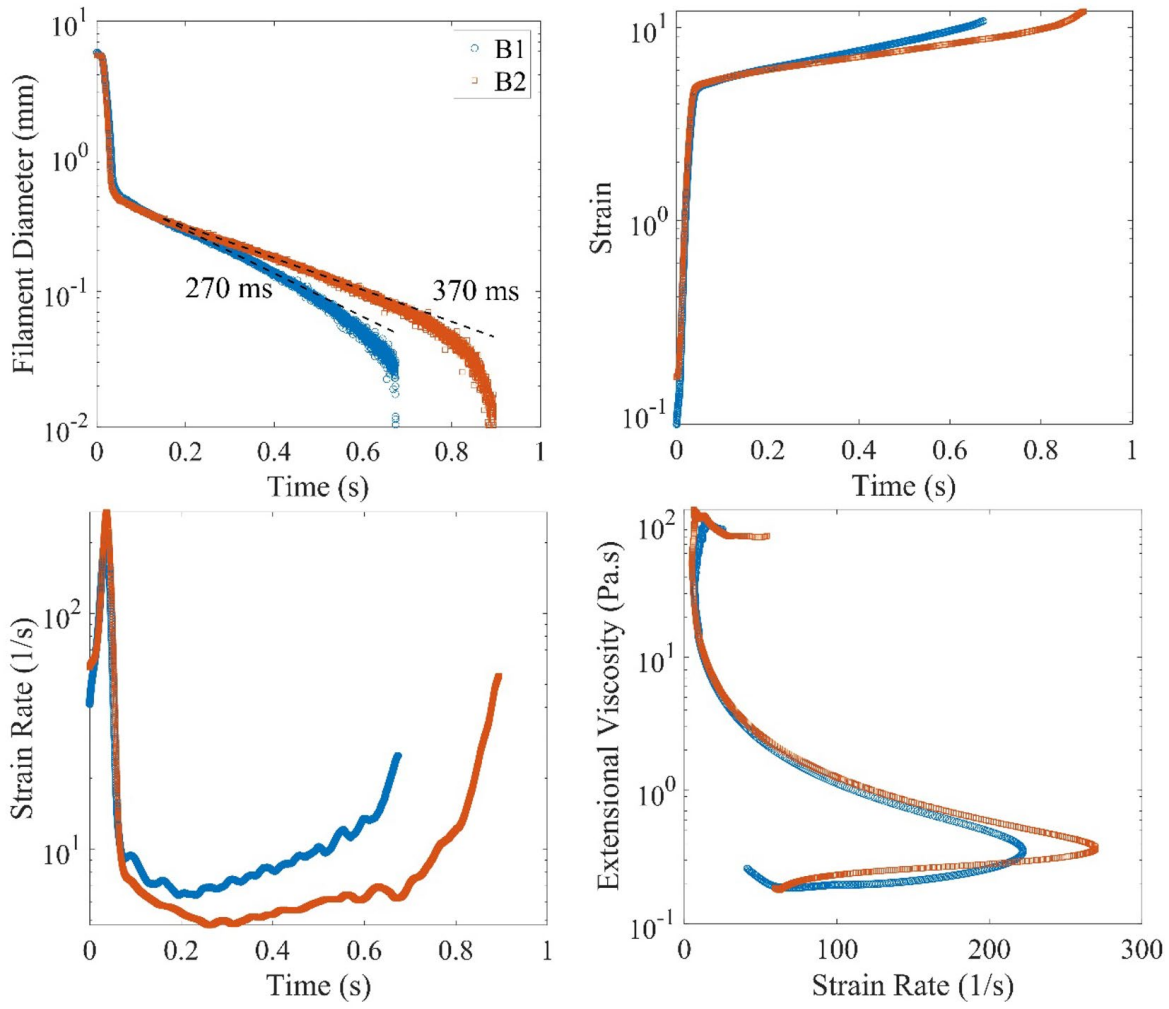
Diagrams of uniaxial-extensional test of polymeric samples at 25 °C.

The extensional viscosity of samples was measured using a HAAKE-CaBER-1 rheometer at the Korean Institute of Science and Technology (KIST). In this test, a droplet of the polymeric sample with a diameter of 6 mm was put between two parallel plates. The upper plate moves rapidly with a linear motor. A linear strike is employed for the motion of the upper plate with a strike time of 50 ms. The extension height is 9 mm, and the Hencky strain is 1.312. The number of data points for the B1 and B2 samples is 7442 and 6735, respectively. The diameter of samples is measured using a laser micrometer. The micrometer's resolution, wavelength, and power are 0.01 mm, 780 nm, and 1.7 mW, respectively. The data from these tests are shown in Fig. 3. According to the figure, the diameter of filaments is decreased during the test, and capillary thinning occurs in the two regimes. At the early time of tests, it is dropped exponentially as^30^:

5 $$d = d_{0} e^{{ - t/\lambda_{c} }}$$

where *d* is the minimum diameter of the filament, $d_{0}$ is the value of *d* at the start of the exponential response, and $\lambda_{c}$ is the characteristic time of thinning. For samples B1 and B2, the values of $\lambda_{c}$ are 270 ms and 370 ms, respectively. In the second regime, the value of *d* deviates from the exponential response, and the polymers are fully stretched. The diagrams of strain and strain rate versus time are also shown in the figure. These graphs could better show the exponential regime because the strain rate is constant in this regime as $\dot{\varepsilon } = 2/\lambda_{c}$. The diagram of extensional viscosity versus the strain rate is also depicted in Fig. 3.

## Governing equations and mathematical formulation

**Figure 4:**
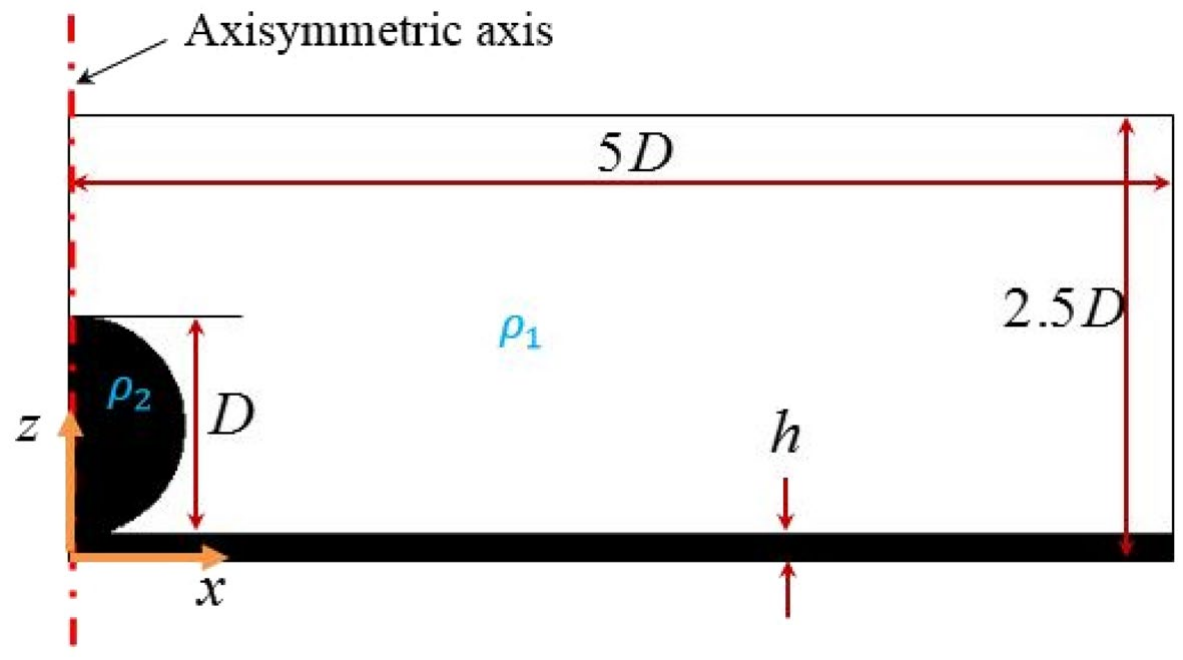
Schematic illustration of drop impact on the liquid film used in our simulations.

A schematic of the drop impact onto the fluid film is portrayed in Fig. 4. The rectangular domain has a length equal to 10*D* and a width equal to 2.5*D*. Moreover, *D*, **g**,* U*_*0*_, ρ_1_, ρ_2,_ and *h* are the drop diameter, the acceleration of gravity, the drop velocity, the densities of fluid one and fluid two, and the film thickness, respectively. In this study, both the drop and liquid film are viscoelastic Boger liquids, while the surrounding air is a Newtonian fluid. Therefore, the equations of motion, i.e., the continuity and momentum equations, for the viscoelastic fluid flow in incompressible and isothermal conditions can be expressed as:

6 $$\nabla \cdot {\text{v}} = 0,$$

7 $$\frac{{\partial \left( {\rho {\text{v}}} \right)}}{\partial t} + \nabla \cdot \left( {\rho {\text{vv}}} \right) = - \nabla p + \nabla \cdot {{\varvec{\uptau}}} + \rho {\text{g}} + {\mathbf{F}}_{s} ,$$

where ${\text{v}}$ is the velocity vector, $p$ is the pressure, $\rho$ is the density, and ${{\varvec{\uptau}}}$ is the stress tensor, which is calculated using the constitutive equation. In Eq. (7), the stress tensor is determined as ${\mathbf{\tau = \tau }}_{{\mathbf{s}}} {\mathbf{ + \tau }}_{{\mathbf{p}}}$, where ${{\varvec{\uptau}}}_{{\mathbf{s}}}$ is the solvent contribution to the stress tensor, and it can be expressed as:

8 $${{\varvec{\uptau}}}_{{\mathbf{s}}} = 2\eta_{s} {\mathbf{D}},$$

where $\eta_{s}$ is the solvent viscosity and ${\mathbf{D}}$ is the deformation rate tensor, defined as ${\mathbf{D}} = \left( {\nabla {\text{v}} + \left( {\nabla {\text{v}}} \right)^{T} } \right)/2$. In this study, the polymer contribution of the stress tensor (${{\varvec{\uptau}}}_{{\mathbf{p}}}$) is obtained from the Oldroyd-B constitutive equation^31^:

9 $${{\varvec{\uptau}}}_{{\mathbf{p}}} + \lambda \mathop {{{\varvec{\uptau}}}_{{\mathbf{p}}} }\limits^{\nabla } = 2\eta_{p} {\mathbf{D}}.$$

where $\lambda$ is the relaxation time, $\eta_{p}$ is the polymeric viscosity at the zero-shear rate, and $\mathop {{{\varvec{\uptau}}}_{{\mathbf{p}}} }\limits^{\nabla }$ is the upper convective time derivate of the stress tensor, defined as:

10 $$\mathop {{{\varvec{\uptau}}}_{{\mathbf{p}}} }\limits^{\nabla } \equiv \frac{{\partial {{\varvec{\uptau}}}_{{\mathbf{p}}} }}{\partial t} + {\text{v}} \cdot \nabla {{\varvec{\uptau}}}_{{\mathbf{p}}} - \left( {\nabla {\text{v}}} \right)^{T} \cdot {{\varvec{\uptau}}}_{{\mathbf{p}}} - {{\varvec{\uptau}}}_{{\mathbf{p}}} \cdot \nabla {\text{v}}.$$

In the above equation, the superscript $T$ is a transpose operator. In the present study, the geometric VOF method is used to treat the interface between the fluids^32^. In Eq. (7), ${\mathbf{F}}_{{\mathbf{s}}}$ denote the surface tension force in the governing equations, which is defined based on the continuous-surface-force (CSF) method, as ${\mathbf{F}}_{{\mathbf{s}}} = \sigma \kappa \nabla \phi$, where $\sigma$ is the surface tension coefficient and $\phi$ is the volume fraction. Also, $\kappa$ introduces the interface curvature of the phases and it can be obtained as follows:

11 $$\kappa = - \nabla \cdot \left( {\frac{\nabla \phi }{{\left| {\nabla \phi } \right|}}} \right).$$

The volume fraction $\left( \phi \right)$ value of each cell can change between zero and one. When a cell is filled with a fluid of phase 1, the $\phi$ value is zero. When a cell is filled with a fluid of phase 2, the $\phi$ value equals 1. Finally, the volume fraction of the fluid interface in the computational cells has a value between 0 to 1 ($0 < \phi < 1$). The shape of the interface is obtained from the following differential equation^33^:

12 $$\frac{\partial \phi }{{\partial t}} + \nabla \cdot \left( {{\text{v}}\phi } \right) + \nabla \cdot \left( {\phi \left( {1 - \phi } \right){\text{v}}_{c} } \right) = 0,$$

where ${\text{v}}_{c}$ is the velocity differences at the interface. The last term in Eq. (12) is considered only at the interface. In the VOF method, the physical and rheological properties of the phases in the control volumes are determined as:

13 $$\zeta = \phi \zeta_{1} + \left( {1 - \phi } \right)\zeta_{2} ,$$

where $\zeta_{1}$ and $\zeta_{2}$ denote the properties of phase 1 and phase 2, respectively.

The non-dimensional parameters employed in this investigation are expressed as follows:

14 $$\begin{gathered} {\text{Re}} = \frac{{\rho U_{0} D}}{{\eta_{0} }},\;We = \frac{{\rho U_{0}^{2} D}}{\sigma },\;Bo = \frac{{\rho gD^{2} }}{\sigma },\;Wi = \lambda \dot{\gamma } = \frac{{\lambda U_{0} }}{D}, \hfill \\ \beta = \frac{{\eta_{p} }}{{\eta_{0} }} = \frac{{\eta_{p} }}{{\eta_{p} + \eta_{s} }},\;H = \frac{h}{D},\;t^{*} = \frac{{U_{0} t}}{D},\;{\text{v}}^{*} = \frac{{\text{v}}}{{U_{0} }},\;{\text{x}}^{*} = \frac{{\text{x}}}{D},\;{\text{z}}^{*} = \frac{{\text{z}}}{D}, \hfill \\ p^{*} = \frac{1}{{\rho U_{0}^{2} }}p,\;\tau^{*} = \frac{1}{{\rho U_{0}^{2} }}\tau, \;\nabla^{*} = D\nabla ,\;{\text{D}}^{*} = \frac{D}{{U_{0} }}{\text{D}}. \hfill \\ \end{gathered}$$

where *Re* is the Reynolds number, *We* is the Weber number, *Bo* is the Bond number, and *Wi* is the Weissenberg number. Furthermore, *β* is the viscosity ratio, *H* is the dimensionless thickness of the liquid film, *U*_*0*_ is the impact velocity, *ρ* is the density of the viscoelastic phase, η_0_ is the viscosity of the viscoelastic phase at the zero-shear rate, σ is surface tension coefficient, *D* is the droplet diameter, and t* is the dimensionless time. The dimensionless forms of the governing and constitutive equations are expressed as follows:

15 $$\nabla^{*} \cdot {\text{v}}^{*} = 0,$$

16 $$\frac{{\partial \left( {{\text{v}}^{*} } \right)}}{{\partial t^{*} }} + \nabla^{*} \cdot \left( {{\text{v}}^{*} {\text{v}}^{*} } \right) = - \nabla^{*} p^{*} + \nabla^{*} \cdot {{\varvec{\uptau}}}^{*} - \frac{Bo}{{We}}\hat{e}_{z} ,$$

17 $${{\varvec{\uptau}}}^{*}_{s} = \frac{2}{{\text{Re}}}\left( {1 - \beta } \right){\mathbf{D}}^{*} ,\;{{\varvec{\uptau}}}^{*}_{{\mathbf{p}}} + Wi\mathop {\tau_{p}^{*} }\limits^{\nabla } = 2\frac{\beta }{{\text{Re}}}{\mathbf{D}}^{*} .$$

### Initial and boundary conditions

According to Fig. 4, the initial and boundary conditions are presented in Eqs. (18) and (19), respectively. Initially, the drop has a downward velocity *U*_*0*_, while the fluid film and the surrounding air are motionless. The no-slip boundary condition is considered at the solid walls. Moreover, the volume fraction gradient, the stress gradient, and the pressure gradient at a direction normal to the walls are set to zero. At the axisymmetric boundary, the gradients of the scalar fields are set to zero. At the top boundary, the atmospheric condition is applied (i.e., for the outflow condition, the parameter gradients are set to zero; for the inflow condition, the parameter values are specified as atmospheric conditions).

18 $$\begin{array}{*{20}c} {\begin{array}{*{20}c} {\rho = \rho_{2} ,y > h} & \to & {\left\{ {\begin{array}{*{20}l} {{\text{v}} = - U_{0} {\hat{\text{j}}}} \hfill \\ {p = 0} \hfill \\ {{\uptau } = 0} \hfill \\ {\phi = 1} \hfill \\ \end{array} ,} \right.} \\ \end{array} } & {\begin{array}{*{20}c} {\begin{array}{*{20}c} {\rho = \rho_{2} ,y \le h} & \to & {\left\{ {\begin{array}{*{20}l} {{\text{v}} = 0} \hfill \\ {p = 0} \hfill \\ {{\uptau } = 0} \hfill \\ {\phi = 1} \hfill \\ \end{array} } \right.} \\ \end{array} ,} & {\begin{array}{*{20}c} {\rho = \rho_{1} } & \to & {\left\{ {\begin{array}{*{20}l} {{\text{v}} = 0} \hfill \\ {p = 0} \hfill \\ {{\uptau } = 0} \hfill \\ {\phi = 0} \hfill \\ \end{array} } \right.} \\ \end{array} } \\ \end{array} } \\ \end{array}$$

At walls:

$$\begin{gathered} \begin{array}{*{20}c} {\nabla p \cdot {\text{n}} = 0,} & {\nabla {\uptau } \cdot {\text{n}} = 0,} \\ \end{array} \hfill \\ \begin{array}{*{20}c} {{\text{v}} = 0,} & {\nabla \phi \cdot {\text{n}} = 0.} \\ \end{array} \hfill \\ \end{gathered}$$

At the vertical axisymmetric boundary:

$$\left\{ \begin{gathered} \begin{array}{*{20}c} {\nabla p \cdot {\text{n}} = 0,} & {\nabla \phi \cdot {\text{n}} = 0,} \\ \end{array} \hfill \\ \begin{array}{*{20}l} {\partial \tau_{xx} /\partial x = \partial \tau_{yy} /\partial x = 0,} \hfill & {\tau_{xy} = \tau_{yx} = 0,} \hfill \\ {\partial v_{y} /\partial x = 0,} \hfill & {v_{x} = 0.} \hfill \\ \end{array} \hfill \\ \end{gathered} \right.$$

At the top boundary:

19 $$\begin{gathered} \rm{Inflow{:}}\begin{array}{*{20}c} {p = 0,} & {{\uptau } = 0,} \\ {{\text{v}} = 0,} & {\phi = 0.} \\ \end{array} \hfill \\ \rm{Outflow{:}}\begin{array}{*{20}c} {\nabla p \cdot {\text{n}} = 0,} & {\nabla {\uptau } \cdot {\text{n}} = 0,} \\ {\nabla {\text{v}} \cdot {\text{n}} = 0,} & {\nabla \phi \cdot {\text{n}} = 0.} \\ \end{array} \hfill \\ \end{gathered}$$

The OpenFOAM software package is employed to discretize and solve the governing equations based on the FVM^34^. The overall algorithm for the isothermal two-phase flow of the viscoelastic flow is based on the Pressure-Implicit with Splitting of Operators (PISO) presented in^27^.

## Results and discussion

**Figure 5:**
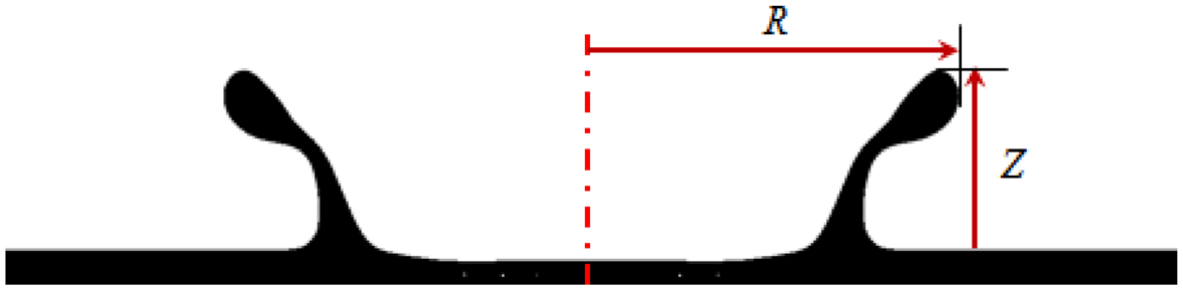
Schematic illustration of crown dimensions.

In this section, the numerical and experimental results will be presented, and the formation and evolution of the crown shape will be investigated in detail. The viscoelastic Boger drop is impacted by the inertia and gravity forces when the drop impacts the same quiescent liquid film. The parameters associated with the crown geometry are the radius (*R*) and the height (*Z*) (refer to Fig. 5).

The dimensionless forms of the radius (*R*) and the height (*Z*) are:

20 $$R^{*} = \frac{R}{D},$$

21 $$Z^{*} = \frac{Z}{D},$$

where $D$ is the drop diameter. It should be noted that the dimensions of the crown are extracted using an image processing technique.

The experiments are performed to investigate the effect of the fluid’s viscoelasticity on the crown dynamics. The experimental results are presented for the different Newtonian and viscoelastic Boger fluids while considering the effects of the drop size, the fluid film thickness, and the drop velocity. In the experimental analysis, the liquid drop is released by its weight and impacts onto the same quiescent liquid film, and this process is recorded via a high-speed camera.

As mentioned in section “Governing equations and mathematical formulation”, the numerical simulation is obtained using the FVM. The numerical study considers specific ranges for the pertinent parameters, including the Weissenberg number (10^−4^ ≤ *Wi* ≤ 10^3^), the Reynolds number (150 ≤ *Re* ≤ 200), the Weber number (200 ≤ *We* ≤ 800), the dimensionless fluid film (0.2 ≤ *H* ≤ 0.35), and the viscosity ratio (0.1 ≤ *β* ≤ 0.5), while the Bond number (*Bo*) is kept constant at *Bo* = 1.2.

### Experimental results

**Figure 6:**
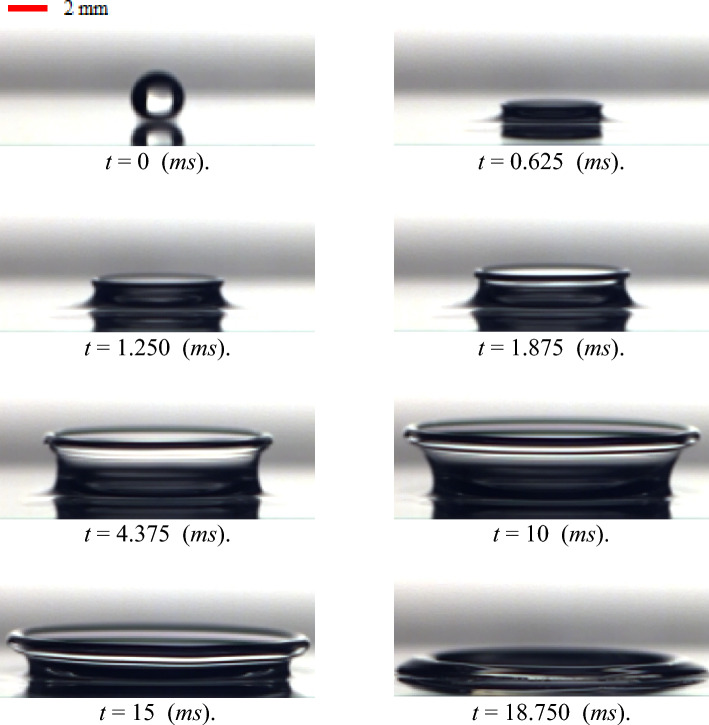
Time sequence of the drop impact onto the liquid film for WG3 for $h =$ 1 mm, $U_{0} =$ 3.92 m/s, *D* = 2.91 mm.

The time sequence of the drop’s impact onto the liquid film is presented in Fig. 6. According to this figure, when the drop comes into contact with the liquid film, the time is set to zero (*t* = 0). Moreover, an image processing technique is utilized to measure the velocity of the droplet prior to its contact with the liquid film. This velocity is used as impact velocity. As the drop impacts onto the liquid film, a liquid jet is ejected from the contact line of the drop and the liquid layer. This jet propagates and spreads with time. According to Fig. 6, the bubbles are also not formed in the present study due to the film’s thin size and the ranges of utilized diameter and velocity of droplets. Some useful information on bubble dynamics is presented in the work of Zhang et al.^35^.

**Figure 7:**
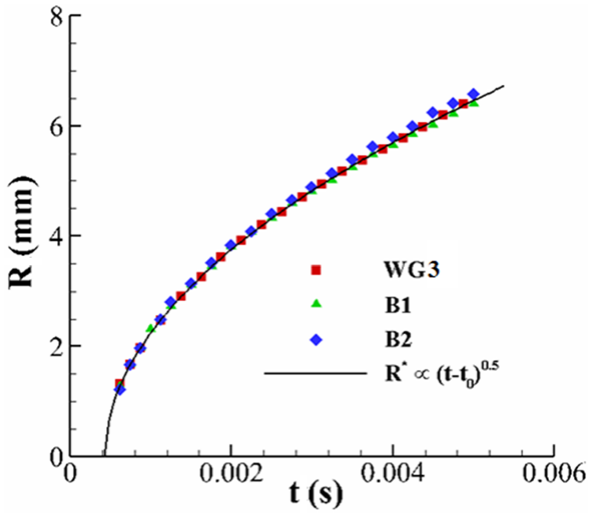
Variation of the crown dimensionless radius with time at the early instance of the impact for $h =$ 1 mm, $U_{0} =$ 3.92 m/s, *D* = 2.91 mm.

Figure 7 demonstrates the time variation of the crown radius for different fluids. According to this figure, the variation of the crown radius with time presents a power-law function. At early instances of the impact, Yarin and Weiss^13^ theoretically predict that the variation of the crown radius is proportional to the square roots of time for Newtonian fluid ($R^{*} \propto \left( {t - t_{0} } \right)^{0.5}$). Our experimental results agree with this prediction at early times. Therefore, at early times, the power-law relation found for Newtonian fluids is also valid for our viscoelastic fluid.

**Figure 8:**
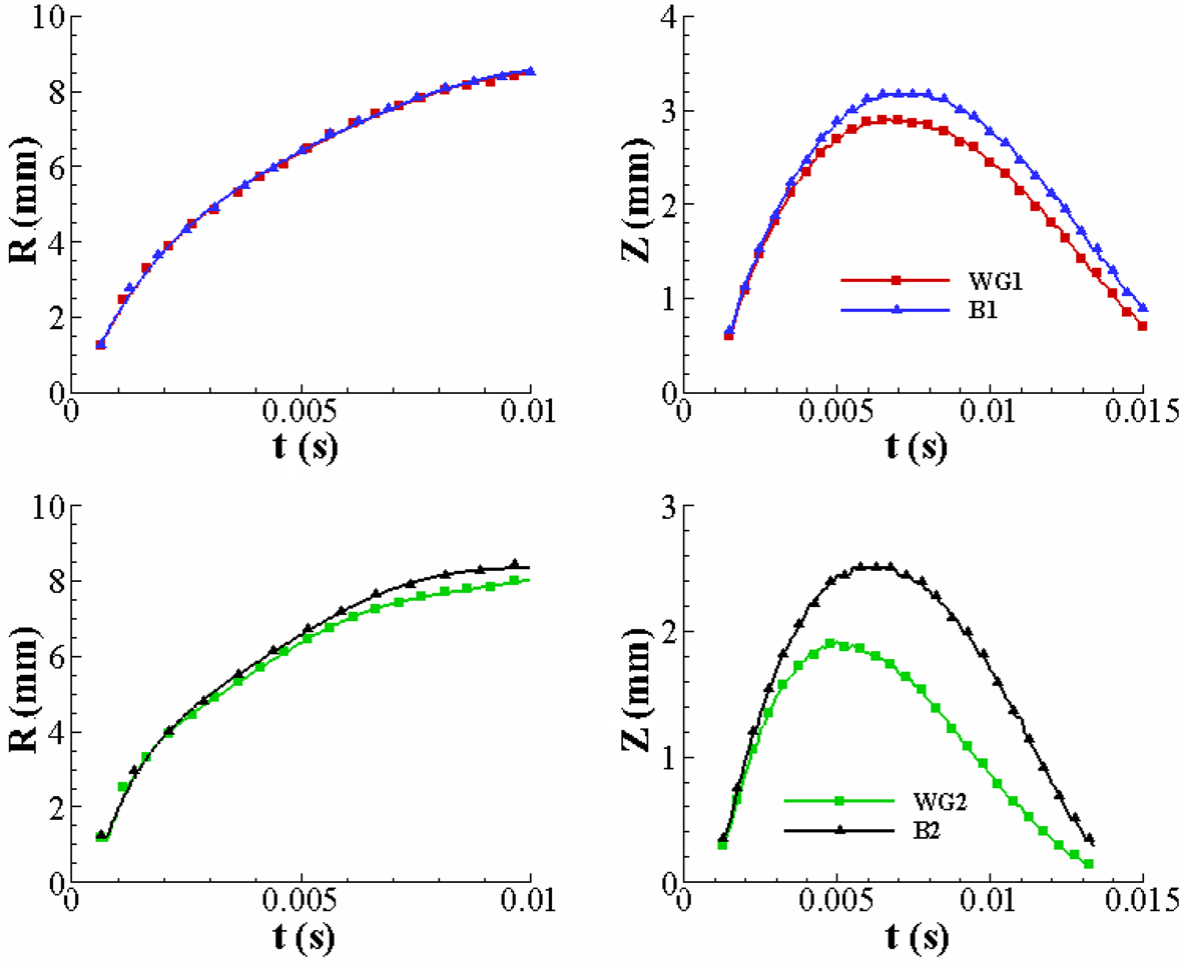
The effect of the fluid’s elasticity on the crown parameters for $h =$ 1 mm, $U_{0} =$ 3.92 m/s, *D* = 2.91 mm.

The effect of the fluid’s elasticity on the time variation of the crown height (Z^*^) and radius (R^*^) is displayed in Fig. 8. In this case, the Newtonian fluids, WG1 and WG2, have a viscosity equivalent to that of the viscoelastic Boger fluids, B1 and B2, respectively. However, the effect of the fluid’s elasticity can be considered individually. Figure 8 indicates that the fluid’s elasticity can enlarge the crown dimension.

**Figure 9:**
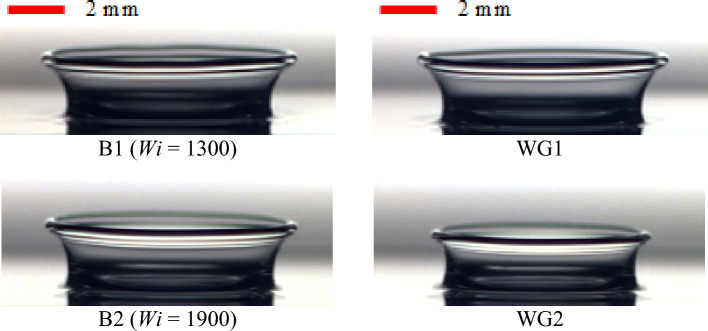
The effect of the fluid’s elasticity on the crown shape parameters for $h =$ 1 mm, $U_{0} =$ 3.92 m/s, *D* = 2.91 mm.

Figure 8 also shows that the effect of the elasticity on the crown height is greater than that on the crown radius. It is clear that by increasing the fluid’s elasticity (using B2 instead of B1), the fluid’s elasticity can enlarge the crown parameters. In other words, the maximum crown height increases by 9.1% and 30.9% when using B1 and B2 instead of the corresponding Newtonian cases. The elastic fluid film can act as an energy conservator that absorbs and then releases the drop kinetic energy during the impact period. Moreover, increasing the fluid’s elasticity diminishes the dissipative energy due to the viscous effect, helping the crown to spread. Figure 9 portrays the crowns shape for different fluids. As a result, by removing the viscosity effect between WG1 and B1, and WG2 and B2, we will witness an increase in the elasticity property leading to the growth of the crown height.

**Figure 10:**
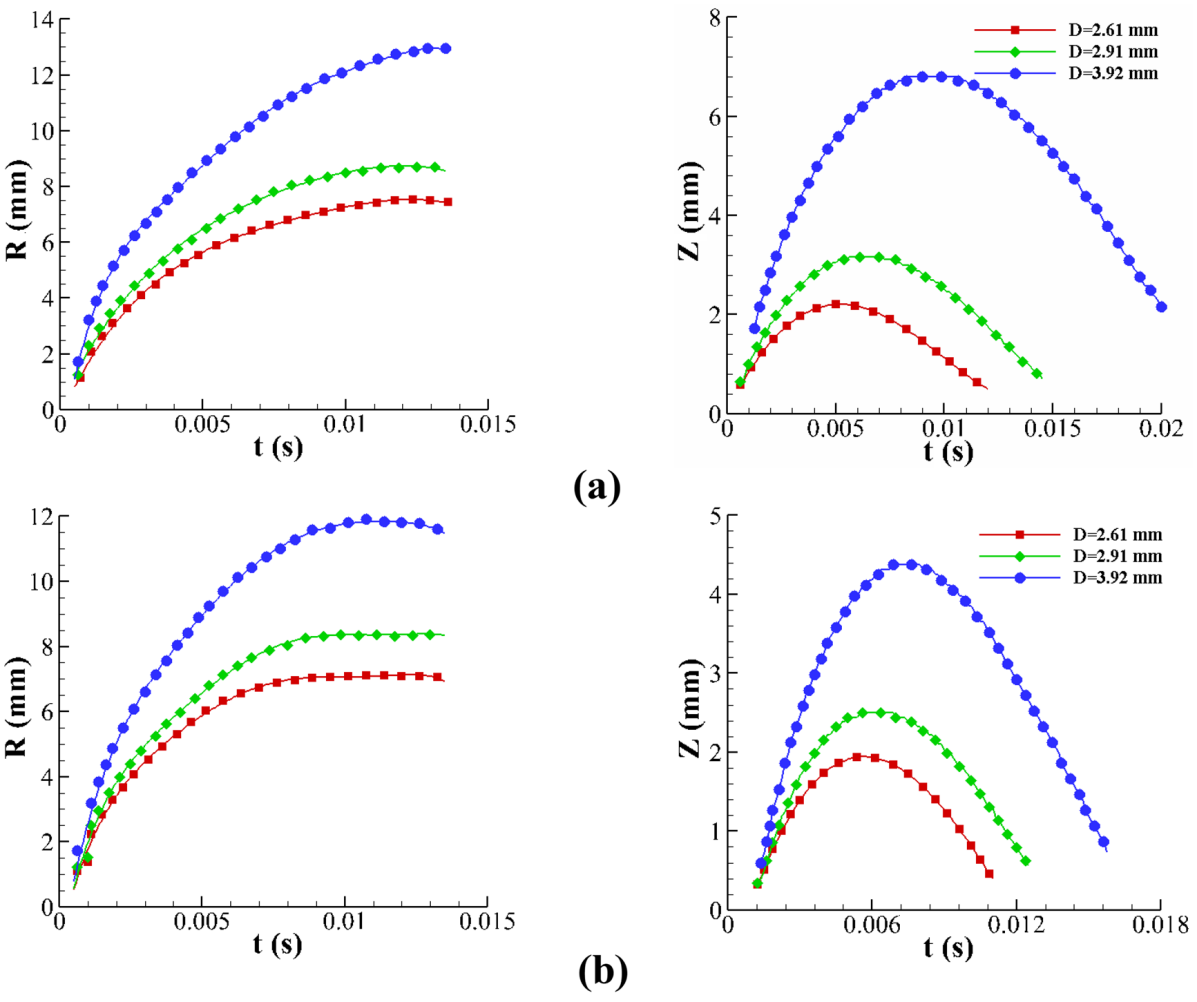
The effect of the drop diameter on the crown parameters for (**a**) B1 and (**b**) B2 for $h =$ 1 mm, $U_{0} =$ 3.92 m/s.

**Figure 11:**
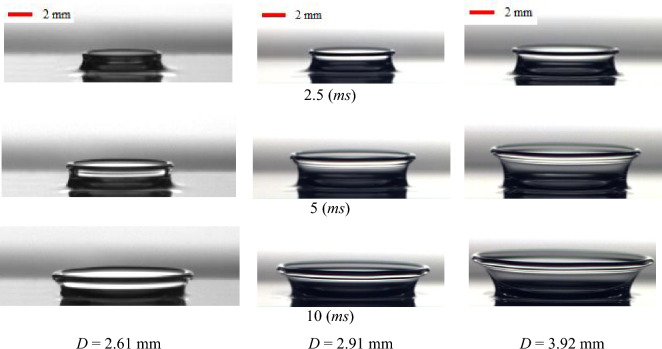
The effect of the drop diameter on the crown shape for B2 for $h =$ 1 mm, $U_{0} =$ 3.92 m/s.

The effect of the drop size on the crown dynamics is reported in Fig. 10. The shape of the drops is close to the sphere. In image processing, the equivalent diameter of the drop is defined as $d_{e} = \sqrt[3]{{d_{h}^{2} d_{v} }}$ where $d_{h}$ and $d_{v}$ are the horizontal and vertical diameters of the drop. As shown in this figure, an increase in the drop diameter causes the growth of the crown dimensions. By increasing the drop size, the kinetic energy and inertia of the drop increase, leading to deeper penetration into the liquid film and spreading a larger amount of the fluid into the crown wall from the liquid film. Moreover, Fig. 10 shows that the influence of the drop size for B1 is greater than that for B2. Quantitatively, by increasing the drop diameter from 2.61 (mm) to 3.92 (mm), the maximum crown height increases by 60% and 30% for B1 and B2, respectively. Using the B2 viscoelastic fluid instead of B1 increases the extensional force in viscoelastic fluid (According to the uniaxial-extensional test of Boger fluids in Fig. 3). However, the extensional force in B2 fluid resists against the spread of crown’s wall. Figure 11 depicts the crown shape for different drop sizes, showing the same trend as in the results of Fig. 10.

**Figure 12:**
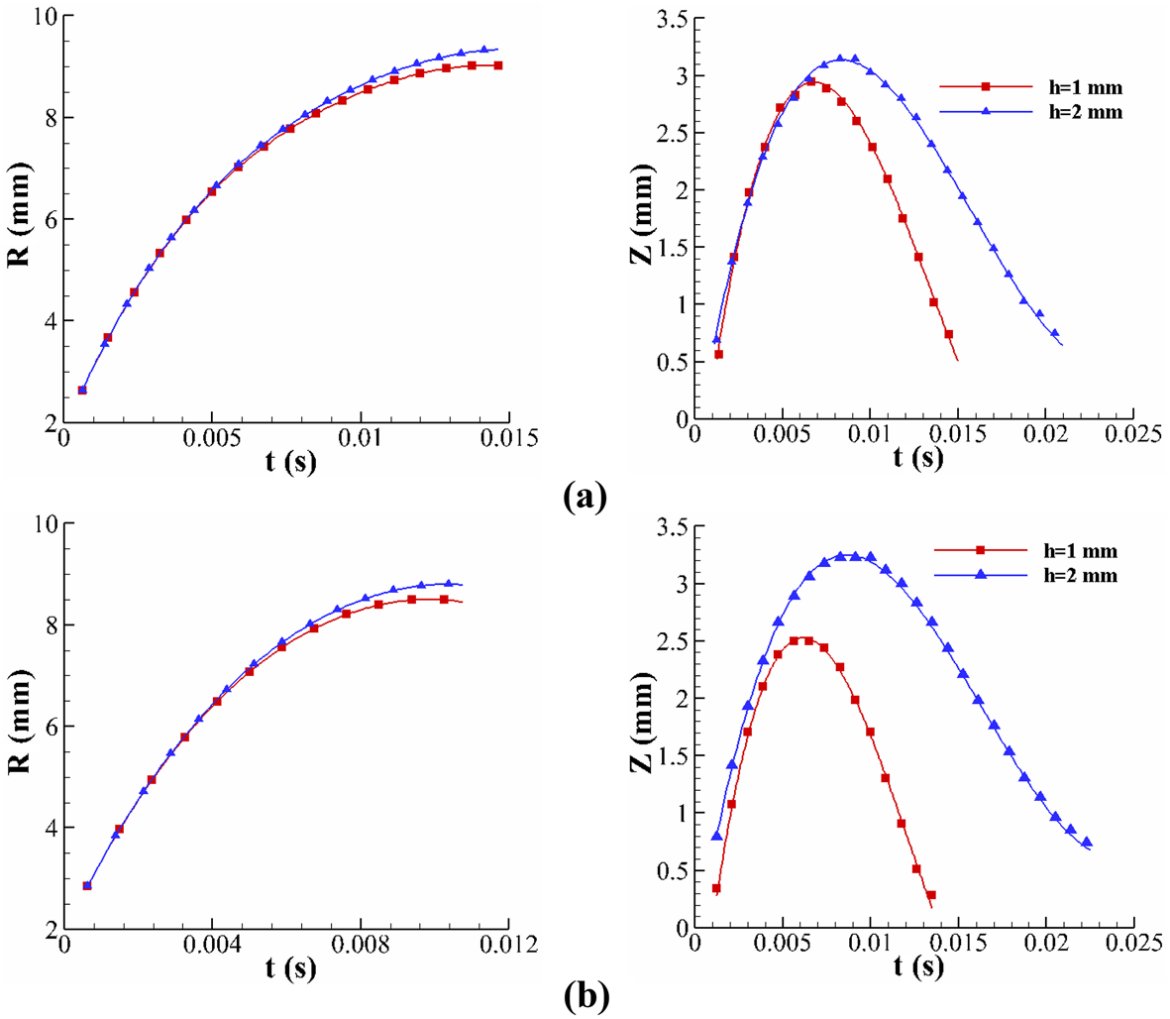
The effect of the film thickness on the crown parameters for (**a**) WG3 and (**b**) B2 for $U_{0} =$ 3.92 m/s, *D* = 2.91 mm.

**Figure 13:**
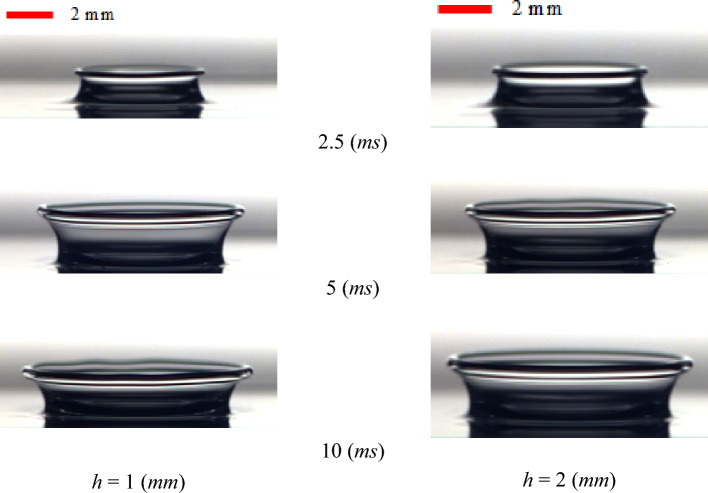
The effect of the film thickness on the crown shape for B2 for $U_{0} =$ 3.92 m/s, *D* = 2.91 mm.

In the impact problem, the liquid film thickness significantly affects the crown dynamics. Figure 12 shows the time variation of the crown dimensions with different film thicknesses in our experimental observation. According to this figure, the crown height grows as the fluid film thickens, in both Newtonian and viscoelastic fluids, while the effect of the film thickness on the crown radius is inconsiderable. Increasing the film thickness means more liquid exits from the liquid layer for the crown to spread. Accordingly, the crown's maximum height increases, and the crown wall's collapse is postponed. An experimental investigation reveals that, for the viscoelastic fluid, the effect of the fluid film thickness on the crown shape is greater; as the film thickness increases from *h* = 1 (mm) to *h* = 2 (mm), the maximum crown height grows by 29% and 6.8% for the Boger and Newtonian fluids, respectively. In the viscoelastic fluid, the elasticity of the fluid film can conserve a greater amount of the impact energy, enhancing the crown dimensions. Moreover, the impact of the fluid film thickness on the crown dynamics is smaller due to the larger amount of viscous dissipation in the Newtonian fluid. The time sequence of the crown shape at different fluid film thicknesses is presented in Fig. 13.

**Figure 14:**
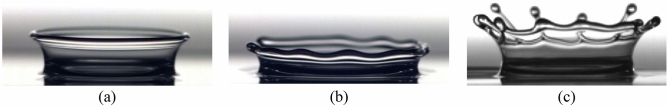
Different regimes in drop impact for B1, (**a**) axisymmetric (multimedia view), (**b**) non-axisymmetric (multimedia view), and (**c**) splashing (multimedia view). The movies of these tests are presented in the supplementary files.

The above experimental results are presented for the axisymmetric case. In our experiments, as the drop’s inertia and the fluid’s elasticity increase and decrease, the axisymmetric form of the crown’s rim may become unstable, perhaps due to Plateau-Rayleigh instability-type instabilities. Therefore, the presentation of our numerical results in the upcoming section (i.e., section “Numerical results”) are limited to the axisymmetric case. An axisymmetric regime corresponding to our experimental results should be determined to validate the numerical results with experiments. In this context, Fig. 14 visually presents the transition between the axisymmetric, non-axisymmetric, and splashing thresholds of the drop impact regimes.

**Figure 15:**
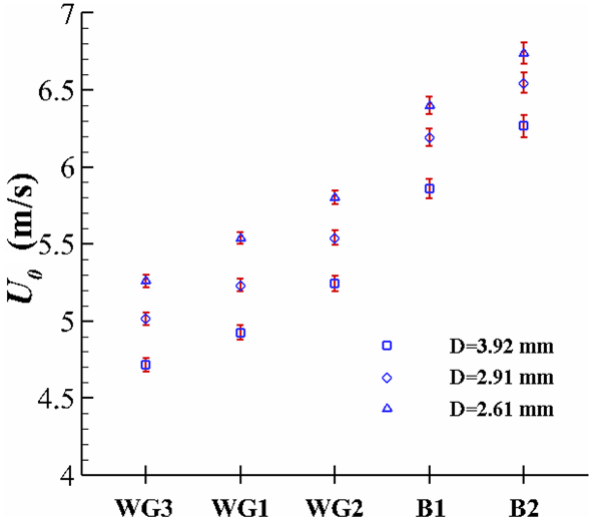
The threshold of the axisymmetric regime breakdown.

In this study, the non-axisymmetric case may appear for a range of drop velocities for different Newtonian and non-Newtonian fluids. For different fluids, the threshold of the drop velocity corresponding to the axisymmetric regime breakdown is presented in Fig. 15. According to this figure, for viscoelastic Boger fluids (B1 and B2), the onset of the regime transition is postponed, due to viscoelastic effects, stabilizing the flow. Moreover, the regime transition occurs at larger drop velocity values in Newtonian fluids by increasing the fluid’s viscosity. Furthermore, larger drop sizes can lead to non-axisymmetric crown shapes for a smaller value of the drop velocity.

### Numerical results

**Figure 16:**
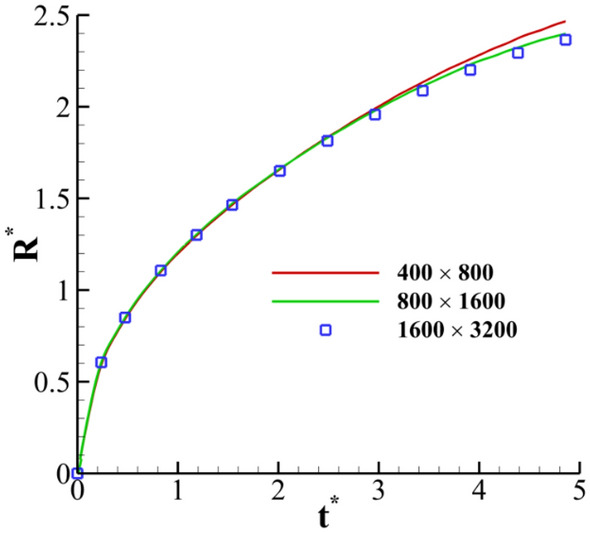
Grid independency analysis for $H =$ 0.2, $Wi =$ 100, *We* = 400, *Re* = 200, and $\beta =$ 0.1.

A grid independence analysis is conducted to ensure our numerical results' accuracy. The variation of the dimensionless crown radius (R^*^) with the dimensionless time is selected as a comparative parameter. Three different grid sizes, i.e., 400 × 800, 800 × 1600, and 1600 × 3200, are used for the grid study. Non-uniform grids are chosen, and these grids are refined near the impact area to better capture the interface between the phases. The variation of the dimensionless radius of the crown (R^*^) with time for three different grid sizes is presented in Fig. 16. The results accuracy increases by increasing the number of grids. This figure also shows that the differences between the results obtained for the grid size of 800 × 1600 and 1600 × 3200 are less than 1%. Moreover, Table 3 reports the maximum value of the stress magnitude with different grid sizes. Table 3 indicates that the numerical error between the grid size of 800 × 1600 and 1600 × 3200 is less than 2.5%; thus, the resolution of 800 × 1600 is selected as the computational grid.

**Table 3:** The variation of maximum value of stress magnitude (*Pa*) with grid size at t^*^ = 3.2 for $H =$ 0.2, $Wi =$ 100, *We* = 400, *Re* = 150, and $\beta =$ 0.1

	Grid size		
	400 × 800	800 × 1600	1600 × 3200
$\left| {{\uptau }_{\max } } \right|$	2.825	3.642	3.739

**Figure 17:**
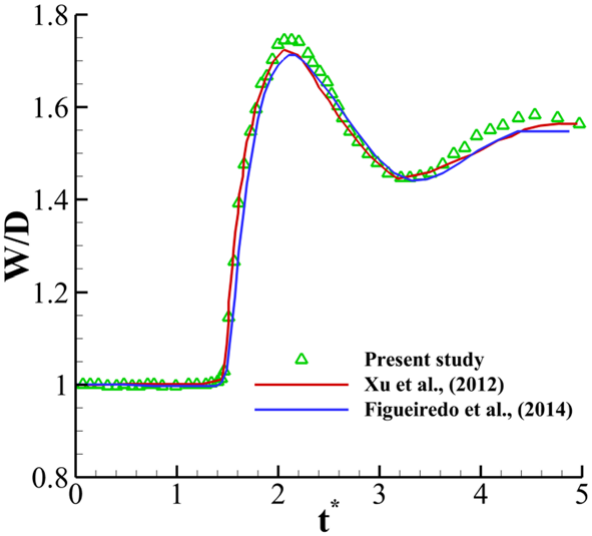
Comparison between the drop width predicted in our work and that in the previous studies^36,37^.

**Figure 18:**
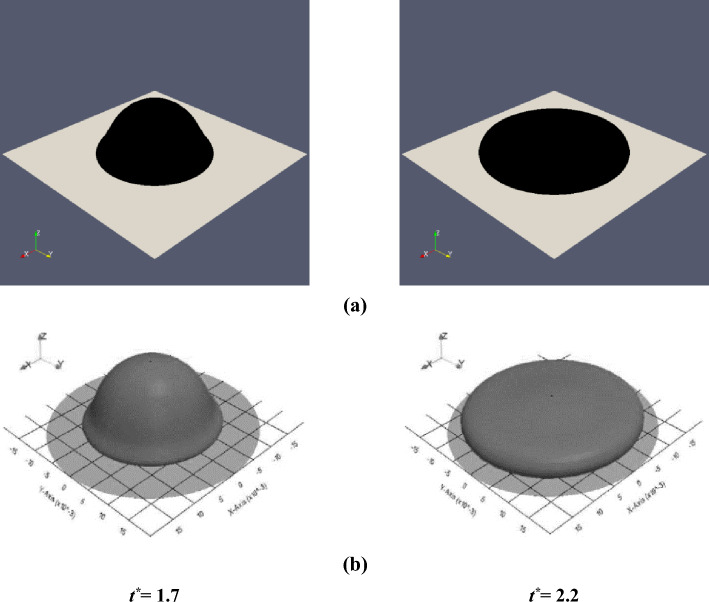
Comparison between the drop shape predicted in (**a**) the present study and (**b**) Figueiredo et al.^37^ for *Re* = 5, *Wi* = 1, *Fr* = 2.26, and *β* = 0.1. Copyright 2016 Elsevier Ltd.

To examine the validity of the numerical solutions, the obtained results are compared with those of the previous numerical studies and the present experimental study. As the first validation case for the axisymmetric solver of the viscoelastic multiphase flow, the impact of the viscoelastic drop onto a dry surface is considered. In this case, a single drop with a diameter of 0.02 (m) is descended under gravity with an initial velocity of 1 (m/s) at a distance of 0.04 (m) from the center of the drop to the rigid drywall. The Oldroyd-B viscoelastic model is selected for the drop phase, and the continuous phase is selected as a Newtonian fluid. In this comparison, the dimensionless parameters are *Re* = 5, *Wi* = 1, *Fr* = 2.26, and *β* = 0.1. Also, the density and viscosity tend to be zero for the Newtonian phase. Here, the results of the present work are verified based on the numerical results of Xu et al.^36^ and Figueiredo et al.^37^, for the width of the drop, as shown in Fig. 17. In Fig. 18, a 3D view of the droplet spreading at different times for the present study and that of Figueiredo et al.^37^ is presented. As can be seen, the current solution for the drop width is in reasonable agreement with the results of the previous studies.

**Figure 19:**
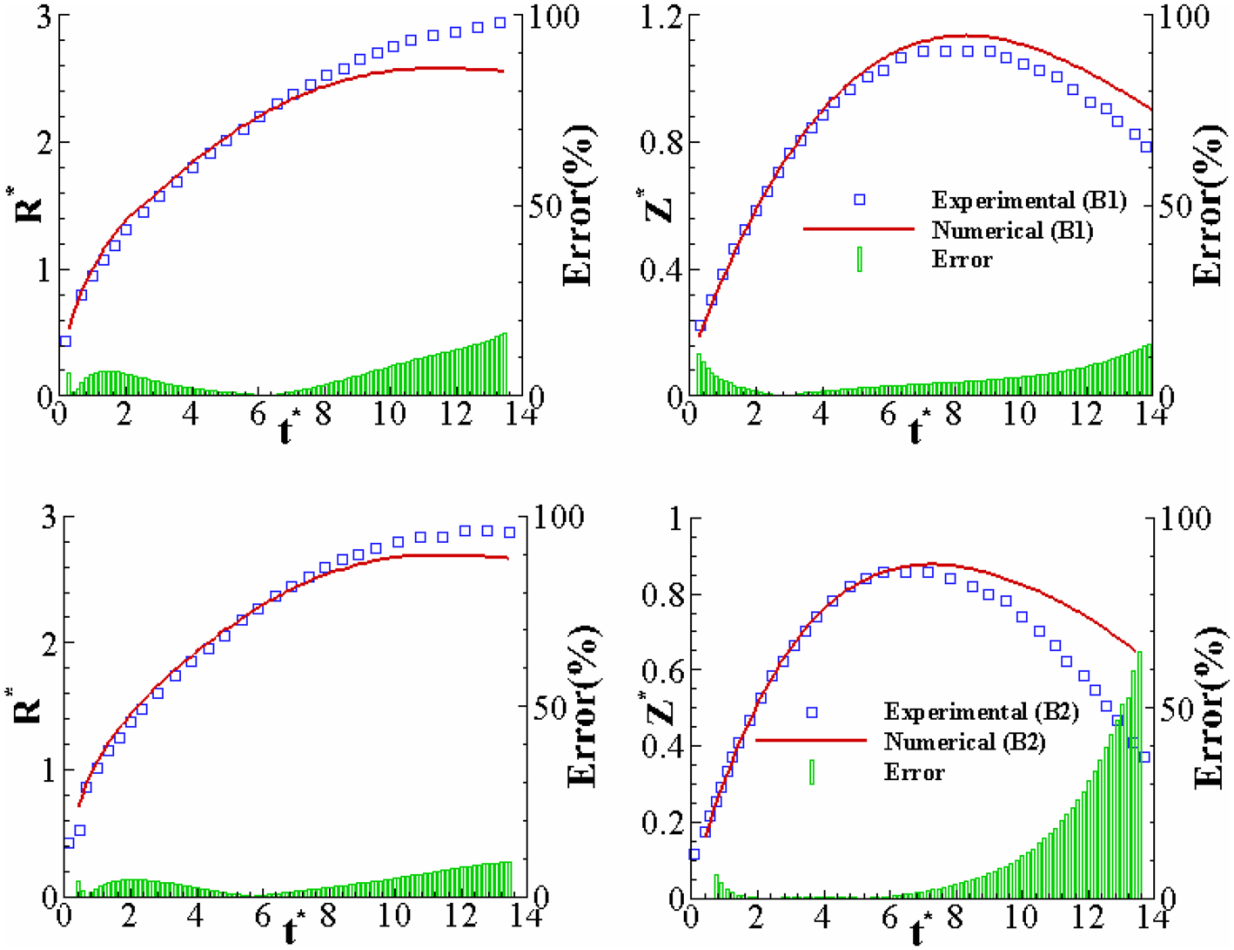
Comparison between the experimental and numerical results of the crown radius and height variations with time for B1 and B2 for *U*_0_ = 3.1 (m/s), *D* = 2.91 (mm) and *h* = 1 (mm).

**Figure 20:**
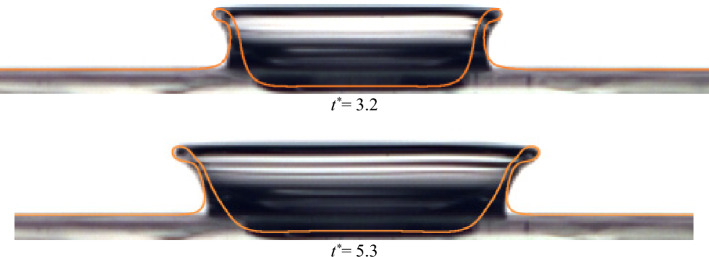
Comparison between the crown shape of B1 at different times for *U*_0_ = 3.1 (m/s), *D* = 2.91 (mm), and *h* = 1 (mm).

Figure 19 compares the current numerical simulation and the experimental data for different viscoelastic Boger fluids (i.e., B1 and B2). Interestingly, the calculated dimensions of the drop (i.e., Z^*^ and R^*^) are in reasonable agreement with the experimental results for small to intermediate times. The numerical and experimental results significantly differ for larger times; this deviation is meaningful for B2. It can be predicted that the source of this error may come from the constitutive equation considered. Also, the crown shape for our numerical and experimental results is illustrated in Fig. 20. The orange line indicates the numerical solution, showing good agreement with the experimental results at different times.

**Figure 21:**
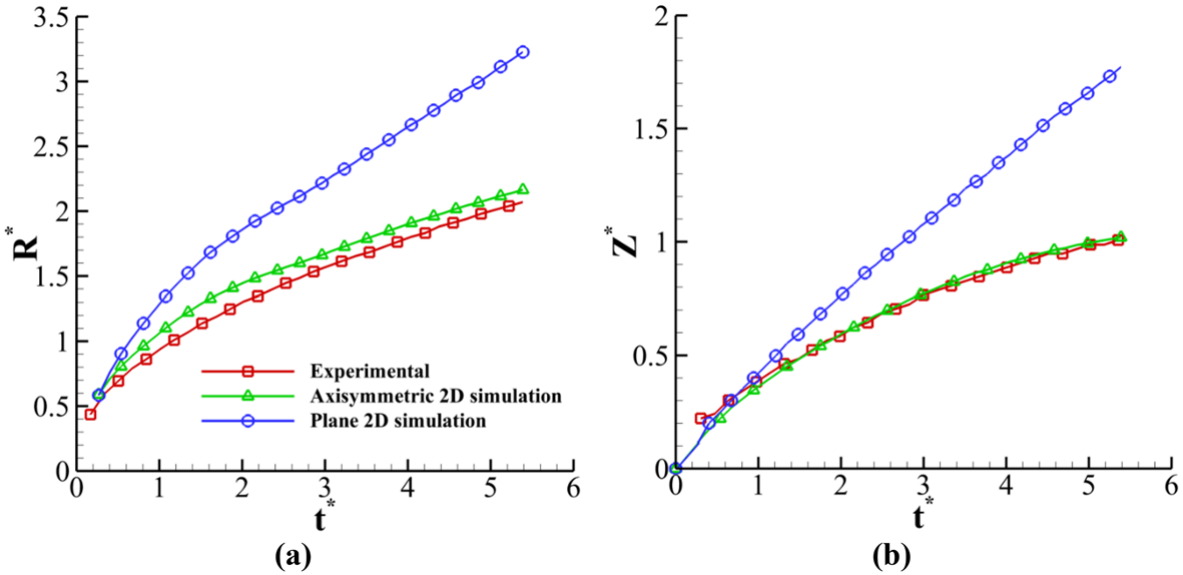
Comparison between numerical (plane two-dimensional^27^ and axisymmetric two-dimensional) and experimental results.

The differences between the plane and axisymmetric two-dimensional simulations and experimental results are represented in Fig. 21. This figure indicates that the axisymmetric results are close to experimental results. In 2D plane simulations, a cylindrical drop spreads a larger amount of fluid from the film layer than the axisymmetric case. Therefore, the present axisymmetric simulation is more accurate than past plane 2D simulations^25^. However, the plane 2D results can be used to recognize drop impact phenomena.

**Figure 22:**
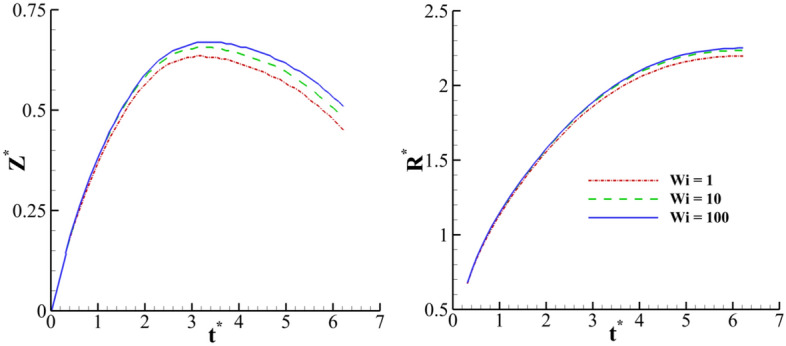
Time variation of the crown dimension with the Weissenberg number for $H =$ 0.2,$\beta =$ 0.1,$Re =$ 175, and $We =$ 400.

**Figure 23:**
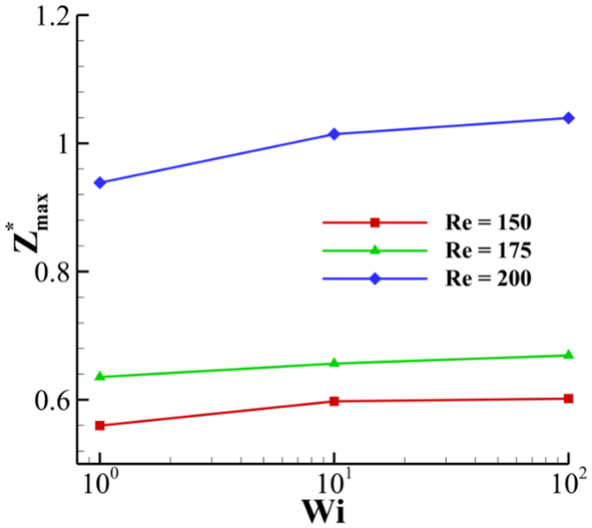
Variation of the maximum crown height with the Weissenberg number at different Reynolds numbers for $H =$ 0.2,$\beta =$ 0.1, and $We =$ 400.

The variation in the crown dimensions, as a function of the dimensionless time, for different Weissenberg numbers are presented in Fig. 22. The Weissenberg number represents the proportion of elastic force to the viscous force in a physical sense. According to this figure, the effect of the fluid’s elasticity on the crown height is more than its effect on the crown radius. In other words, by varying the Weissenberg number from 1 to 100, at t^*^ = 6, the dimensionless crown height (Z^*^) and the crown radius (R^*^) increase by 11.5% and 2.3%, respectively. Furthermore, Fig. 22 indicates that the fluid’s elasticity causes a simultaneous increment in both the crown height and the radius. This figure also shows that the maximum value of the crown height is increased by enhancing the fluid’s elasticity. Therefore, increasing the Weissenberg number increases the elasticity of fluid. In the early instances of the impact, the fluid’s elasticity of the liquid film can absorb the drop inertia. Consequently, at the stage of the crown spreading, the stored energy of elasticity in the fluid film can be converted to kinetic energy. According to Fig. 22 and 23, the Weissenberg number is important in the impact problem.

**Figure 24:**
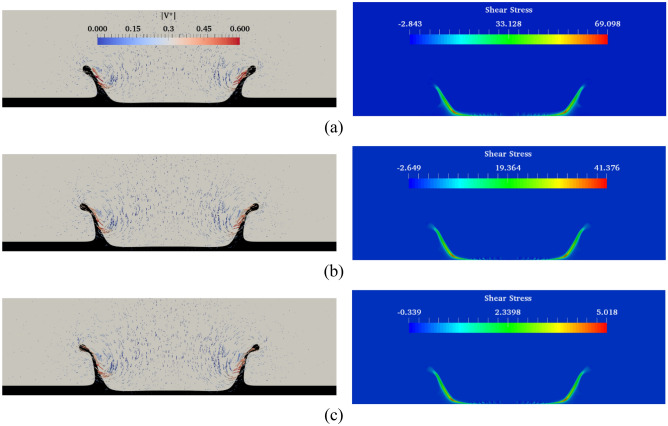
Crown shape and shear stress fields at different Weissenberg numbers at t^*^ = 2.8 for $H =$ 0.2,$\beta =$ 0.1, and $We =$ 400 (**a**) $Wi =$ 1, (**b**) $Wi =$ 10, and (**c**) $Wi =$ 100.

The effect of the Reynolds number on the maximum crown height at different Weissenberg numbers is illustrated in Fig. 23. The Reynolds number physically shows the ratio of inertia force to the viscous force. This figure shows that, as the inertial force is strengthened, it can amplify the drop’s kinetic energy, and the crown’s maximum height is also enlarged. In addition, Fig. 23 indicates that the elasticity has the same role on the crown dimension at the different Reynolds numbers. The effect of the Weissenberg number on the crown shape at t^*^ = 2.8 is demonstrated in Fig. 24. As shown in Fig. 24, the crown thickness decreases, and the crown wall is stretched by enhancing the fluid’s elasticity. This figure also shows the velocity vector field surrounding the impact area at different Weissenberg numbers. Due to the interaction between the crown and the surrounding air, the vortices are formed near the middle of the crown wall. According to Fig. 24, the maximum value of the velocity field decreases by increasing the Weissenberg number. At larger values of *Wi,* the crown reaches its maximum height earlier than the case at lower values of *Wi.*

The effect of the Weissenberg number on the magnitude of the shear stress field is also shown in Fig. 24. According to Fig. 24, the maximum value of the shear stress is located in the base of the crown. The results indicate that the maximum value of the shear stress field decreases as the Weissenberg number grows. By enhancing the fluid’s elasticity, a higher percentage of the drop’s kinetic energy is converted to the crown due to the energy restoration capability of the fluid film. In contrast, by decreasing the Weissenberg number, the energy dissipation is increased over the impact period. As it is known, the energy loss by viscous dissipation depends strongly on the shear stress. As seen in Fig. 24, decreasing the Weissenberg number increases the maximum value of the shear stress field, leading to an augmentation in the dissipative energy and a reduction in the crown dimensions.

**Figure 25:**
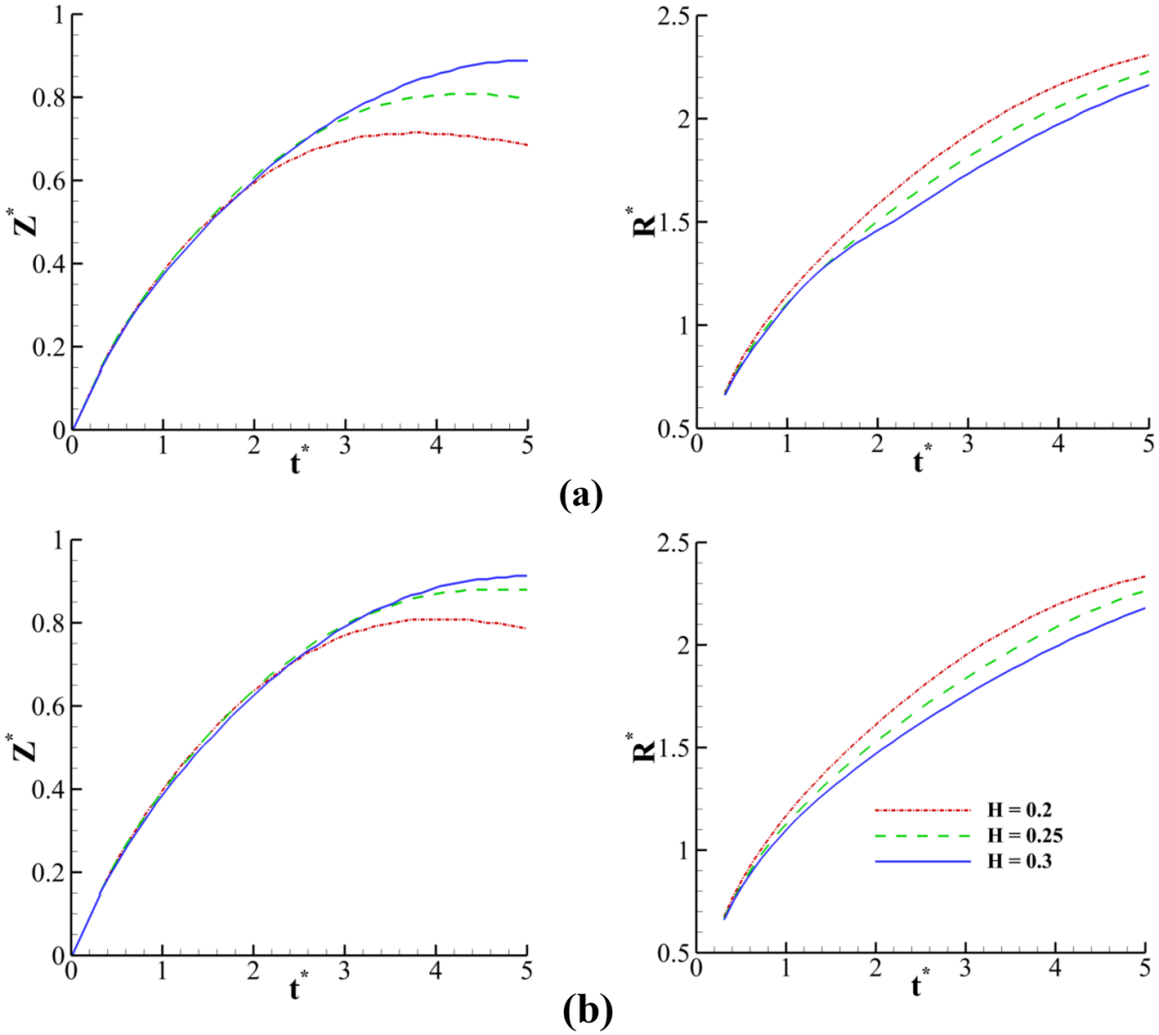
Time variation of crown radius and height with film thickness for $Re =$ 175,$\beta =$ 0.1, and $We =$ 554 (**a**) $Wi =$ 1 and (**b**) $Wi =$ 100.

**Figure 26:**
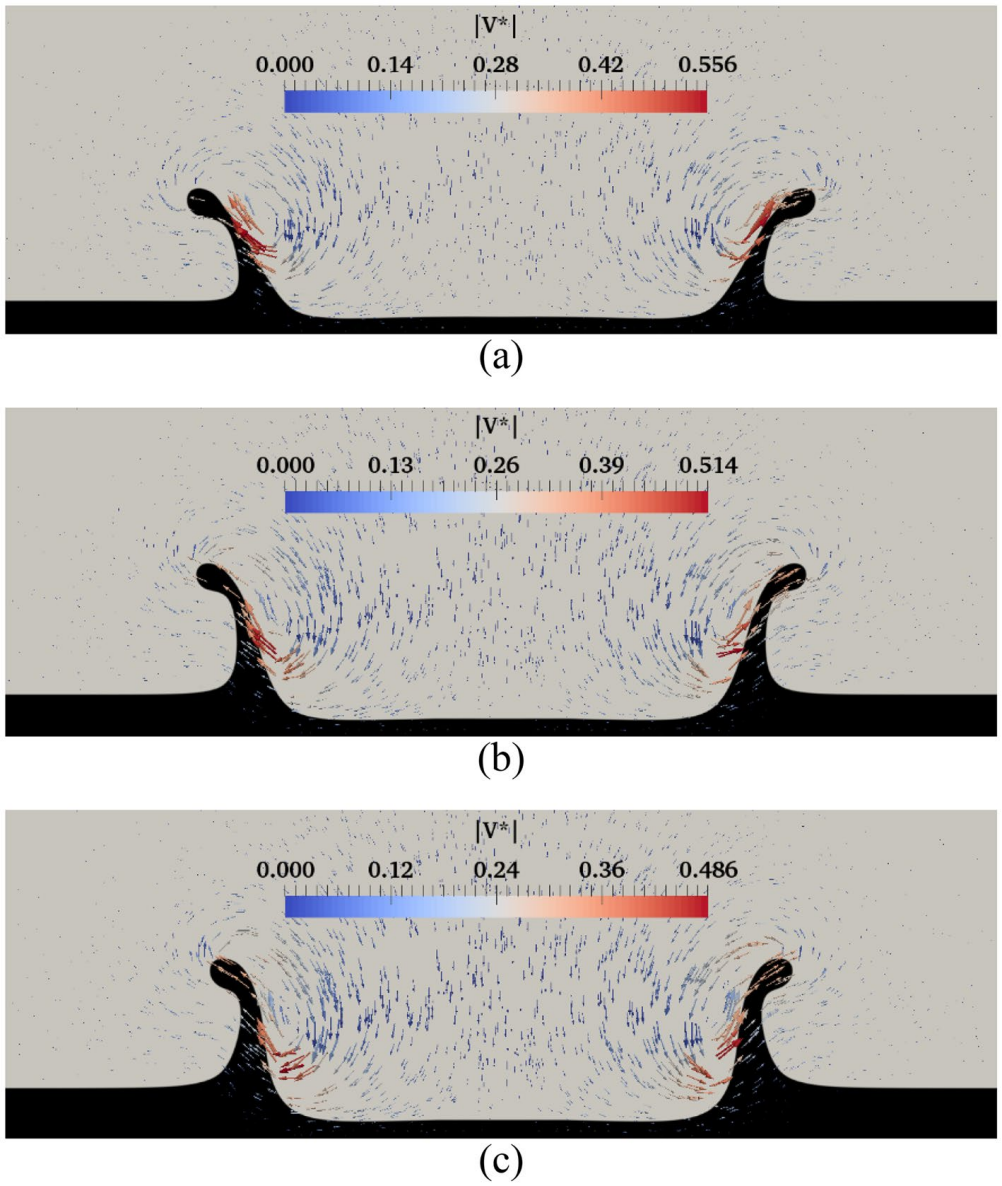
Variation of the crown shape with the film thickness at t^*^ = 2.8 for $Wi =$ 100, $\beta =$ 0.3, $Re =$ 175, and $We =$ 544 (**a**) $H =$ 0.2, (**b**) $H =$ 0.25, and (**c**) $H =$ 0.3.

Figure 25 demonstrates the time variation of the crown dimension with the fluid film thicknesses (*H*) at different Weissenberg numbers. As the fluid film thickens, the crown radius decreases, and the crown height increases at different times. By increasing the fluid film thickness, the kinetic energy of the drop is used to penetrate into the thicker liquid film that opposes the radial movement of the fluid. Moreover, as the film thickness increases, the elastic fluid film recovers a higher portion of the impact energy of the drop, leading to an increase in the vertical elevation of the crown. According to Fig. 25, the elasticity can promote the rate of increase of Z^*^. At t^*^ = 2.8, the effect of the film thickness on the crown shape and the velocity field is depicted in Fig. 26. According to this figure, in a thin film, the fluid motion is primarily in the radial direction, while in a thick film, the fluid motion is generally in the direction normal to the fluid film. In other words, increasing the liquid film thickness enlarges the angle of the crown wall.

**Figure 27:**
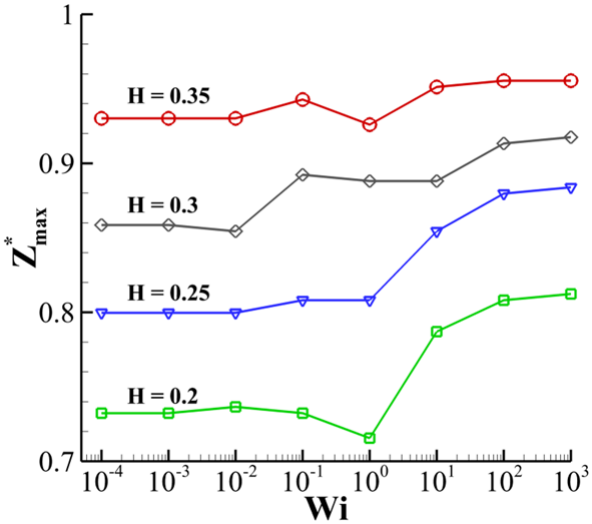
Variation of the maximum crown height with the Weissenberg number at different fluid film thicknesses for $Re =$ 175,$\beta =$ 0.1, and $We =$ 400.

In Fig. 27, the effect of the fluid film thickness on the maximum height of the crown is presented at different Weissenberg numbers. With an increase in the fluid film thickness, the maximum height of the crown ($Z_{\max }^{*}$) increases. Overall, by increasing the Weissenberg number from 10^–4^ (almost a Newtonian fluid) to larger values of 10^3^, the maximum height increases. With an increase in *Wi*, i.e., at higher $H$, the rate of increase of $Z_{\max }^{*}$ decreases. According to this figure, the maximum crown height has complex behavior around *Wi* = 1. In the viscoelastic flow, the dimensionless form of the extensional viscosity (Trouton ratio) is defined as follows^38^:

22 $$\frac{{\overline{\eta }}}{{\eta_{0} }} = \frac{{\tau_{xx} - \tau_{yy} }}{{\eta_{0} \dot{\varepsilon }}}.$$

**Figure 28:**
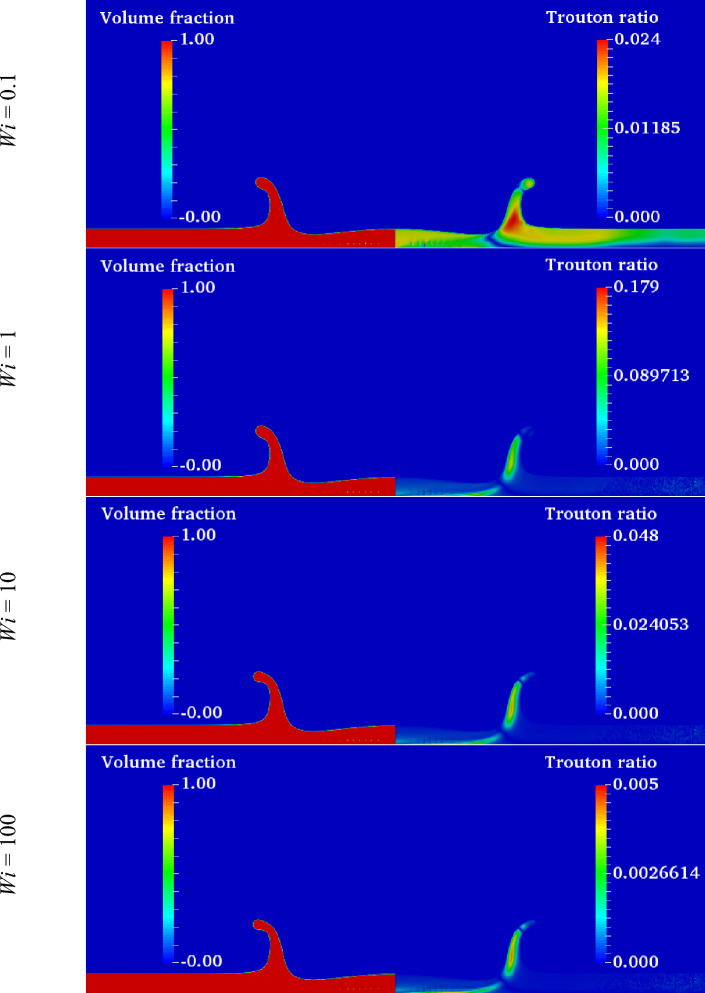
Contours of the volume fraction and Trouton ratio at different Weissenberg numbers at t^*^ = 1.6 for $Re =$ 175,$\beta =$ 0.1, and $We =$ 400.

As seen in Fig. 28, the maximum value of the Trouton ratio is located in the crown wall. At *Wi* ≈ 1, the extensional viscosity has a maximum value in the range of *Wi* = 0.1–*Wi* = 100. Therefore, the extensional force prevents the growth of the crown height at moderate Weissenberg numbers (*Wi* = *0.1 … 1*). On the other hand, as the Weissenberg number is increased further to 1000, a higher portion of the impact energy can be conserved by the fluid film. The conserved elastic energy helps to increase the maximum crown height at higher values of the Weissenberg number.

**Figure 29:**
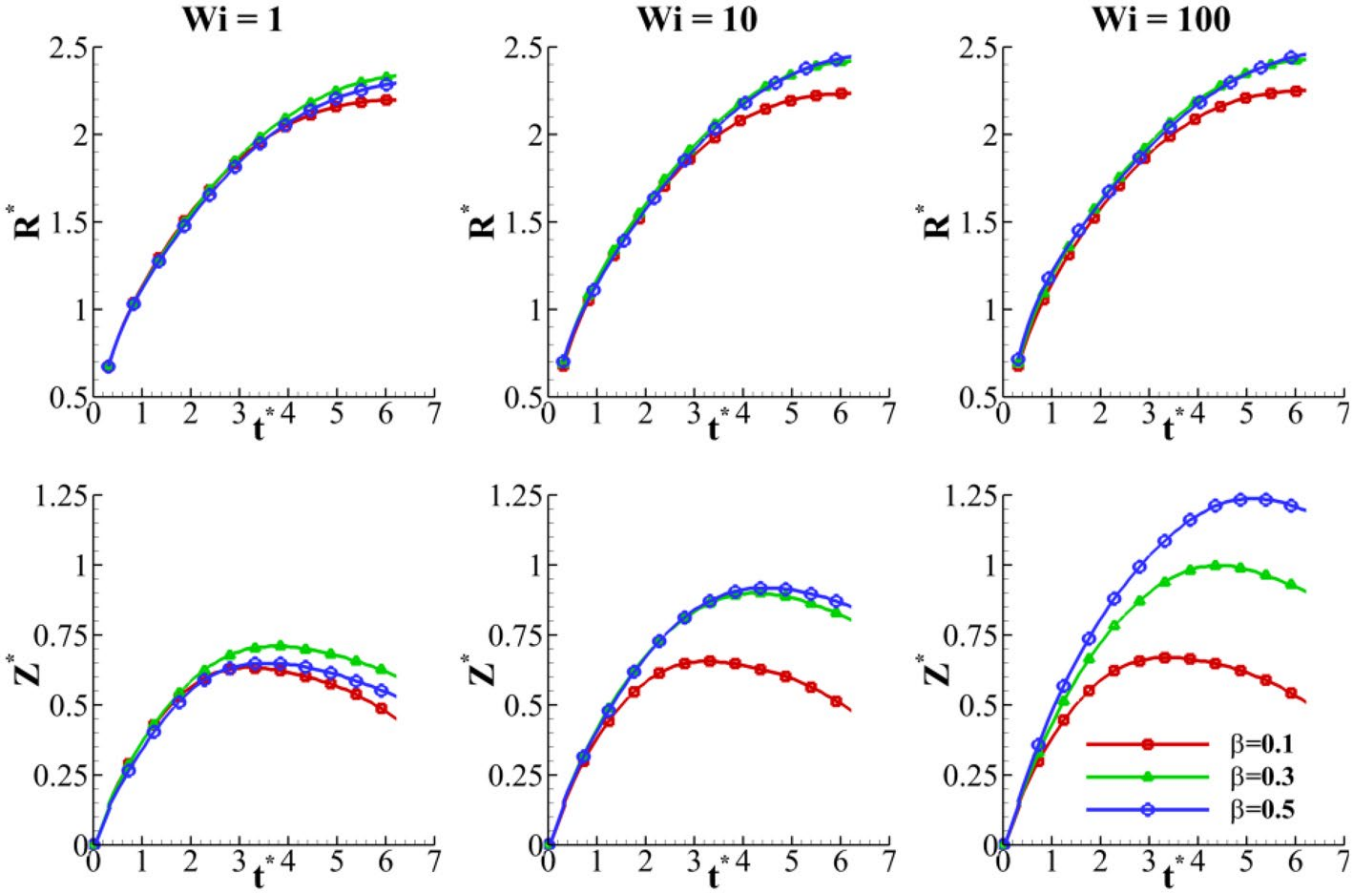
Time variation of crown radius and height with viscosity ratio for $Re =$ 175, $H =$ 0.2, and $We =$ 400.

Figure 29 depicts the effects of the viscosity ratio ($\beta$) on the crown dynamics at different Weissenberg numbers. The viscosity ratio introduces the ratio of the polymeric contribution of the viscosity to the solution’s viscosity at zero shear rate ($\beta = {{\eta_{p} } \mathord{\left/ {\vphantom {{\eta_{p} } {\eta_{0} }}} \right. \kern-0pt} {\eta_{0} }}$). The results are presented in terms of $\beta$, but note that $\eta_{0}$ is fixed. As shown in Fig. 29, the effect of the viscosity ratio on the crown height is more considerable than that on the crown radius. Moreover, at larger *Wi*, the crown height increases by increasing the viscosity ratio. This is due to a higher polymer viscosity in viscoelastic solution, causing to enhances the elasticity of the polymeric solution. Hence, the effect of $\beta$ on the crown height is similar to that of the Weissenberg number.

**Figure 30:**
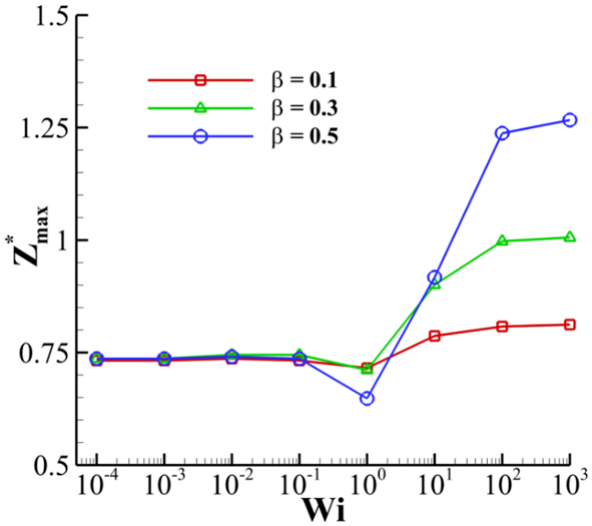
Variation of the maximum crown height with the Weissenberg number at different viscosity ratios for $Re =$ 175,$H =$ 0.2, and $We =$ 400.

**Figure 31:**
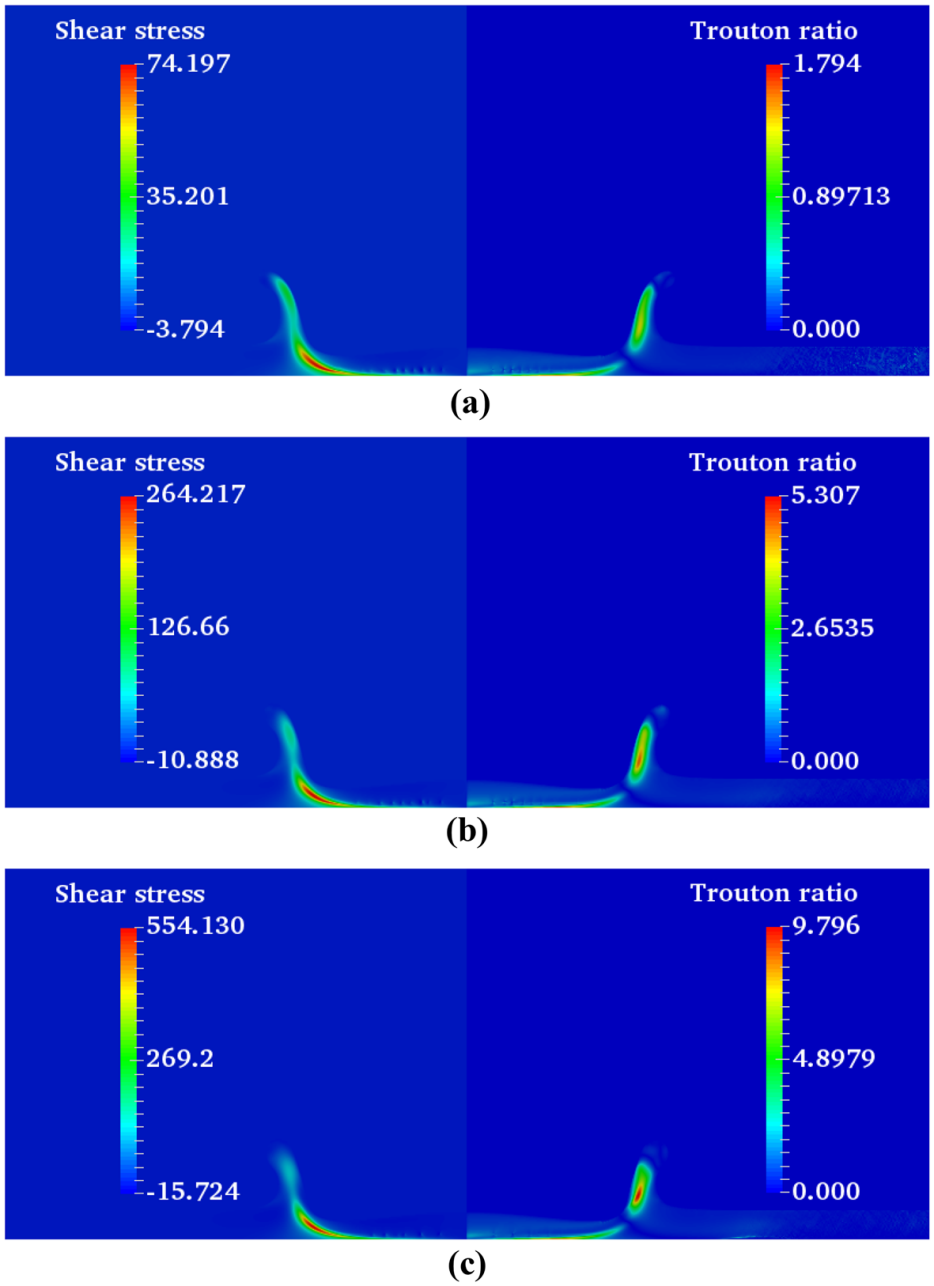
Shear stress and Trouton ratio field at different viscosity ratio at t^*^ = 1.6 for $H =$ 0.2, $Wi =$ 1, and $We =$ 400 (**a**) $\beta =$ 0.1, (**b**) $\beta =$ 0.3, and (**c**) $\beta =$ 0.5.

**Figure 32:**
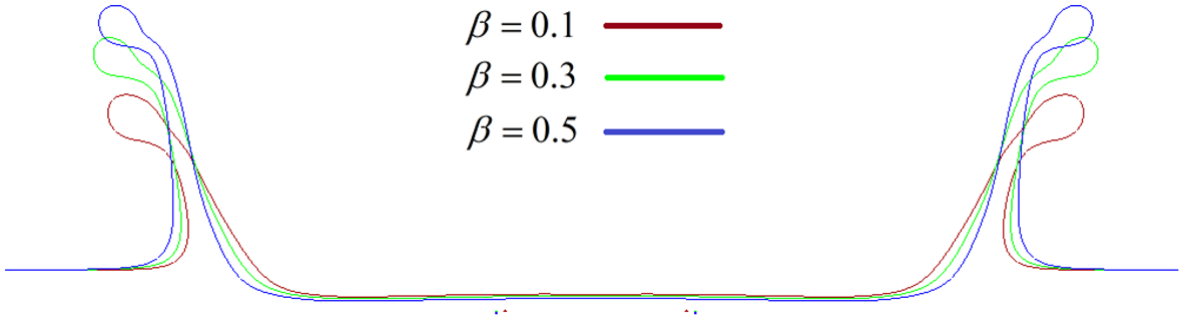
Variation of the crown shape with the viscosity ratio at t^*^ = 2.8 for $Wi =$ 100, $H =$ 0.2, $Re =$ 175, and $We =$ 400.

On the other hand, at lower *Wi*, the influence of the viscosity ratio on the crown characteristics is negligible. The maximum crown height is compared at three viscosity ratios of 0.1, 0.3, and 0.5 in Fig. 30. According to this figure, at larger values of the Weissenberg number, the effect of the viscosity ratio on the $Z_{\max }^{*}$ is significant. It can be seen that for a large value of the Weissenberg number (*Wi* ≥ 10), the crown maximum height is increased as the viscosity ratio increases. As *Wi* is decreased (*Wi* ≤ 0.1), the variation of $Z_{\max }^{*}$ with *β* converges to the same value and, therefore, it becomes invariable to the viscosity ratio. In elastic flows with small values of the Weissenberg number (*Wi* ≤ 0.1), the viscoelastic constitutive equation is insensitive to the variation of the viscosity ratio. On the other hand, for high elastic flows (*Wi* ≥ 10), *β* has a major effect on the viscoelastic flow that enhances the elasticity of the polymeric solution. As seen in Fig. 30, the maximum crown height changes trends at *Wi* ≈ 1. According to Fig. 31, increasing the viscosity ratio increases the fluid’s elasticity and extensional force in the crown wall. This can control the propagation of the crown; at *Wi* = 1, a larger amount of the impact energy is dissipated with respect to large Weissenberg numbers. Therefore, it is clear that, at moderate Weissenberg numbers (*Wi* ≈ 1), the viscosity ratio parameter can suppress the crown spreading. The effect of the viscosity ratio on the crown shape is displayed in Fig. 32 when t^*^ = 2.8.

**Figure 33:**
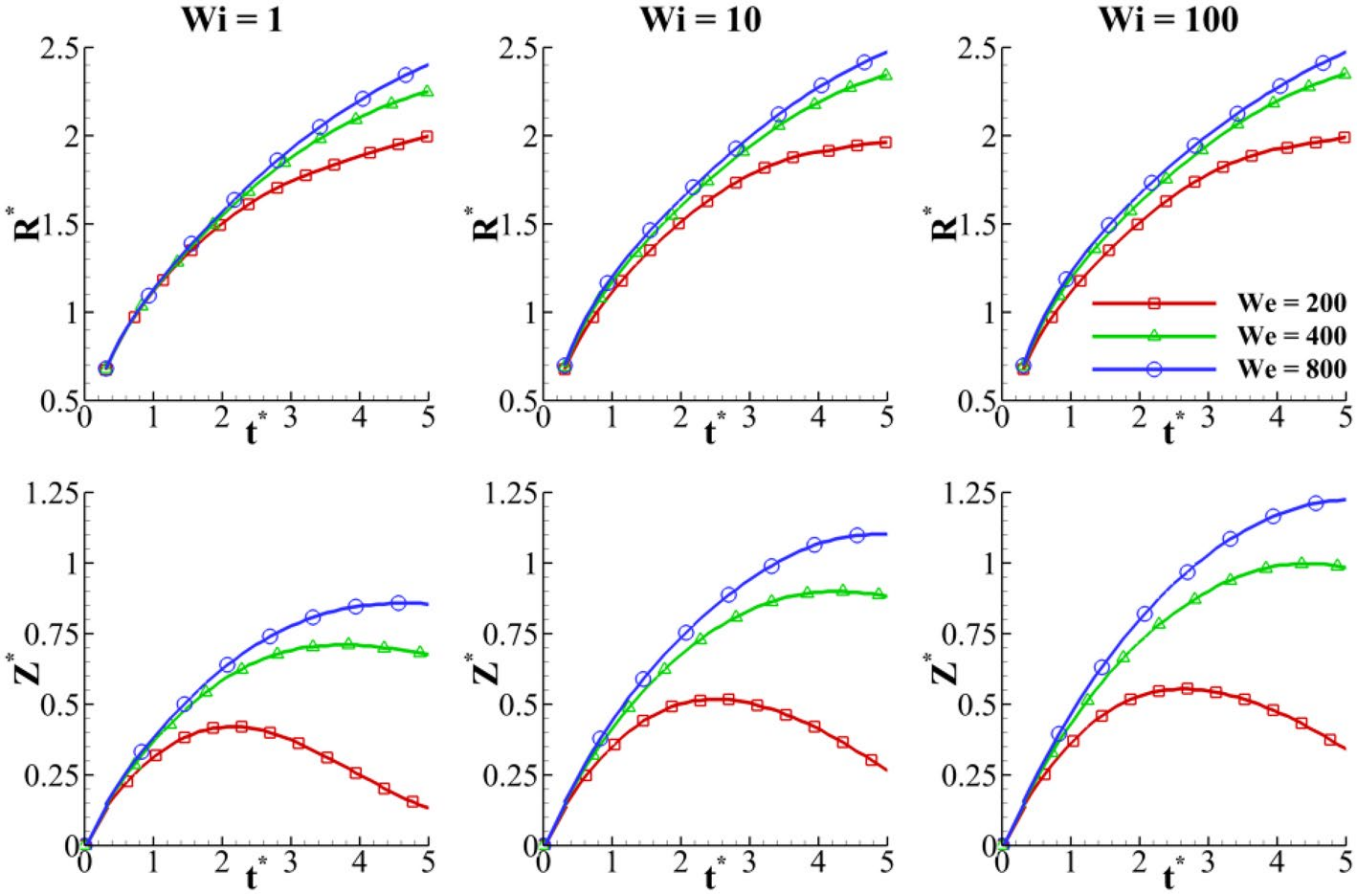
Time variation of the crown radius and height with the Weber number for $Re =$ 175, $H =$ 0.2, and $\beta =$ 0.3

Figure 33 compares the crown dimensions (Z^*^ and R^*^) at different Weber and Weissenberg numbers. The results are reported for a range of Weber numbers between *We* = 200 and *We* = 800, at different Weissenberg numbers. The Weber number shows the ratio of the inertia to the surface tension force. As it can be seen from Fig. 33, by increasing the Weber number, the crown height and radius increase. This figure also shows that the fluid’s elasticity has negligible effects on the variation of the crown radius at different Weber numbers. In contrast, this figure indicates that the Weissenberg number significantly influences the variation of the crown height at different Weber numbers. Moreover, as the Weber number varies between 200 and 800, at t^*^ = 5, the parameter of Z^*^ increases 555%, 328% and 273% for *Wi* = 1, *Wi* = 10 and *Wi* = 100, respectively. As a result, by increasing the fluid’s elasticity, the rate of increase of Z^*^ decreases.

**Figure 34:**
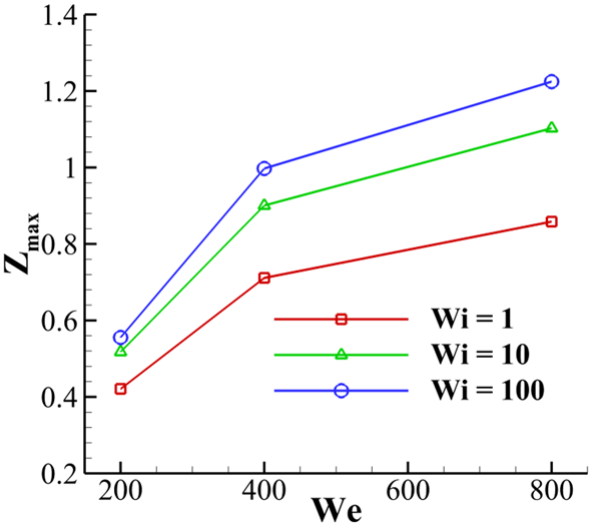
Variation of the maximum crown height with Weissenberg number at different viscosity ratios for $Re =$ 175,$H =$ 0.2, and $\beta =$ 0.3.

Figure 34 depicts the variation of the maximum crown height in terms of the Weissenberg and Weber numbers. As the Weber number increases, the Z^*^ parameter increases at different Weissenberg numbers. Moreover, as the Weissenberg number increases, the Weber number strongly influences Z^*^. In this study, the inertia force increases by increasing the drop velocity, leading to the dominance of the inertial force over the surface tension force. As a result, the drop inertial force increases the size of the crown. When the drop momentum is increased, the liquid layer spreads out more rapidly, and the crown can expand to greater heights. Moreover, Fig. 34 reveals that the collapse of the crown wall is delayed as the Weber number increases.

**Figure 35:**
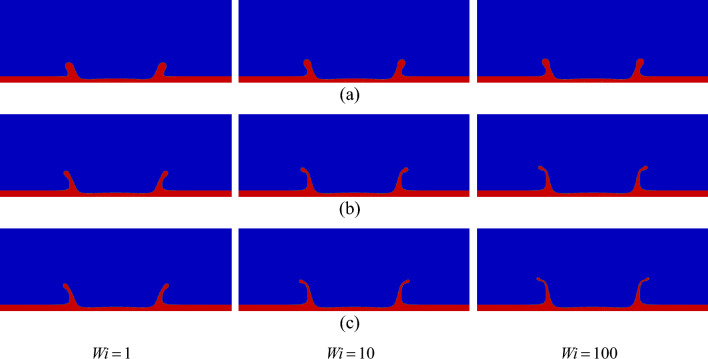
Variation in the crown shape with the Weber number at t^*^ = 2.8 for $\beta =$ 0.3, and *H* = 0.2, (**a**) $We =$ 200, (**b**) $We =$ 400, and (**c**) $We =$ 800.

The shape of the crown at different Weber and Weissenberg numbers is illustrated in Fig. 35. According to Fig. 35, for a large value of the Weber number (*We* = 800), the crown wall is thinner, whereas for a lower Weber number (*We* = 200), the surface tension force resists the enlargement of the crown wall; therefore, the body of the crown wall is thicker.

## Conclusions

Numerical and experimental investigations are conducted to understand the effect of a fluid’s viscoelasticity on the crown formation and propagation due to a drop impact onto a liquid film. Firstly, an experimental study (using high-speed imaging) is performed to investigate the effect of the relevant parameters on the impact problem. Also, the validity of the present numerical investigation is evaluated/confirmed by comparing the results with both the present experimental observation and the past numerical studies. In the numerical part, the governing equations are solved using a two-dimensional axisymmetric solver based on the FVM. The Oldroyd-B model was considered as the constitutive equation. The VOF technique was applied to track the interface between the phases.

In the experimental part, the effects of the drop diameter, the film thickness and the drop velocity are mainly considered. To analyze the crown dynamics via experiments, the crown height and radius variation are presented versus the aforementioned parameters. The experimental results indicate that increasing the fluid’s elasticity can increase the crown wall height. Also, the crown dimensions increase by increasing the drop size, the fluid film thickness and the drop velocity (Supplementary file).

In the numerical part, the effects of the Weissenberg number, the viscosity ratio, the Weber number, and the dimensionless film thickness on the flow field are investigated. The results of the numerical investigation show that the fluid’s elasticity can increase the crown dimensions significantly for small values of the film thickness (*H* ≈ 0.2), whereas, at large values of the film thickness (*H* ≈ 0.35), the crown dynamics have not affected severely from the fluid’s elasticity. By decreasing the Weissenberg number, the maximum value of the shear stress increases in the crown base, which dissipates the drop's initial kinetic energy. In contrast, as the Weissenberg number increases, the kinetic energy of the drop is conserved during the impact period. Moreover, the results indicate that, at the moderate Weissenberg numbers (0.1 ≤ *Wi* ≤ 1), the extensional force plays an important role in the dynamics of the crown, controlling the crown propagation. The results also indicate that, by increasing the fluid film thickness, the rate of increase of the crown height increases, while the time variation of the crown radius decreases. The influence of the viscosity ratio on the crown dynamics strongly depends on the Weissenberg number. For highly elastic flows (*Wi* ≥ 10), the viscosity ratio helps to enlarge the crown dimensions, whereas, for intermediate values of the Weissenberg number (0.1 ≤ *Wi* ≤ 1), the viscosity ratio suppresses the crown propagation. It is found that the Weber number has a significant impact on the crown dimensions. Increasing the Weber number increases the time variation of the crown parameters due to the weakening of the surface tension force. The results also indicate that the effect of Weber number on crown dynamics is more significant for larger values of the Weissenberg number. It is worth mentioning that the impact of a viscoelastic drop onto an elastic substrate would constitute a cognizant route for future work^39^.

## Supplementary Information

Supplementary Video 1.

Supplementary Video 2.

Supplementary Video 3.

## List of symbols

$$Bo$$: Bond number
$$D$$: Drop diameter
$g$: Acceleration of gravity
$$h$$: Film thickness
$H$: Dimensionless film thickness
$$p$$: Pressure
$$R$$: Crown radius
$${\text{Re}}$$: Reynolds number
$$t$$: Time
$$U_{0}$$: Drop velocity
$${\text{v}}$$: Velocity vector
$$We$$: Weber number
$$Wi$$: Weissenberg number
*x*: *x*- Coordinate
*z*: *z*- Coordinate
$$Z$$: Crown height

## Greek symbols

$$\alpha$$: Mobility factor
$$\beta$$: Viscosity ratio
$$\phi$$: Volume fraction
$$\eta$$: Viscosity
$$\kappa$$: Interface curvature
$$\lambda$$: Relaxation time
$$\rho$$: Density
$$\sigma$$: Surface tension
$$\tau$$: Stress

## Superscripts and subscripts

$$p$$: Polymer
$$s$$: Solvent
